# Supplementary material for: Guided phase transition for mitigating voltage hysteresis of iron fluoride positive electrodes in lithium-ion batteries
Source: Nat Commun. 2025 Sep 29;16:8596. doi: 10.1038/s41467-025-63676-9 (PMC12479885; doi:10.1038/s41467-025-63676-9)
Supplement: Supplementary file 1 — Supplementary Information [file 41467_2025_63676_MOESM1_ESM.pdf]

Supporting Information for

Guided phase transition for mitigating voltage hysteresis of iron fluoride positive electrodes in lithium-ion batteries

Hyoi Jo,[‡1] Minjeong Gong[‡ 2], Se Young Kim[3], Dong-Hwa Seo[*2] & Sung-Kyun Jung[*1,4,5,6]

Affiliation

[1]Institute for Battery Research Innovation, Seoul National University, Seoul, Republic of Korea.

[2]Department of Materials Science and Engineering, Korea Advanced Institute of Science and Technology (KAIST), Daejeon, Republic of Korea.

[3]Energy Storage Research Center, Korea Institute of Science and Technology (KIST), Seoul, Republic of Korea.

[4]Department of Materials Science and Engineering, College of Engineering, Seoul National University, Seoul, Republic of Korea.

[5]School of Transdisciplinary Innovations, Seoul National University, Seoul, Republic of Korea.

[6]Research Institute of Advanced Materials, Seoul National University, Seoul, Republic of Korea.

‡ These authors contributed equally: Hyoi Jo, Minjeong Gong

* These authors jointly supervised this work: Dong-Hwa Seo, Sung-Kyun Jung (email: dseo@kaist.ac.kr and naecard@snu.ac.kr)

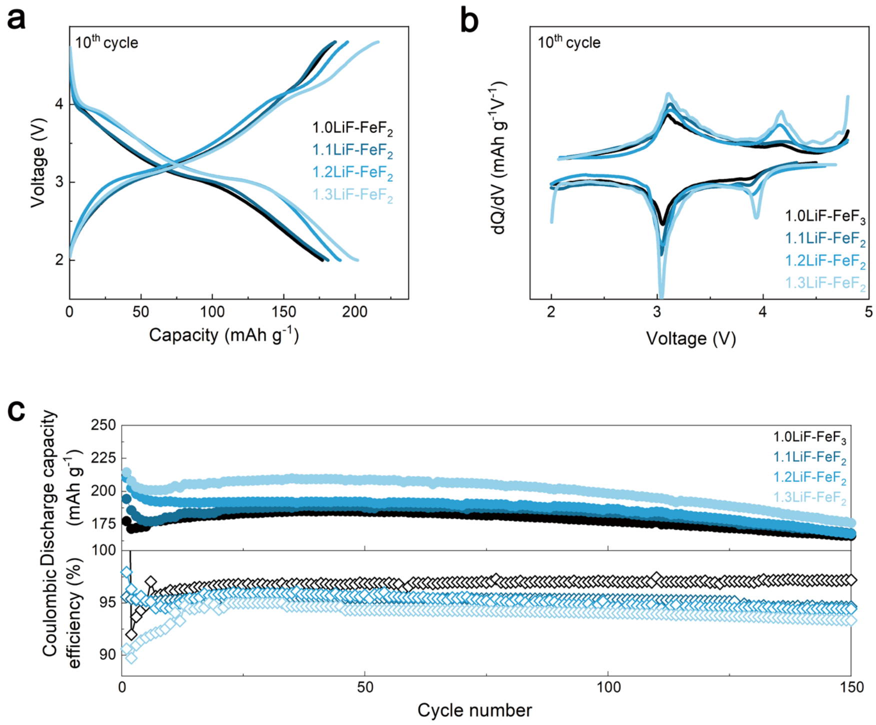

Supplementary Figure S1: Effect of LiF Content on Electrochemical Performance. (a-c) Comparison of 10th charge/discharge profiles, differential analysis of voltage profiles, and cycle performance for various amounts of LiF at 25 °C and 20 mA g^{-1} current density.

Supplementary Note 1. Comparison of Electrochemical Profiles Based on LiF Content in LiF-FeF_2 Nanocomposites

The electrochemical performance of LiF-FeF_2 nanocomposites with varying molar ratios of LiF was systematically evaluated (Supplementary Figure S1). An increase in the LiF content corresponded with a notable enhancement in capacity, as well as a more pronounced plateau at 4V. This phenomenon is likely due to the augmented formation of new polymorphs resulting from the increased interaction between LiF and FeF_2. Nevertheless, when the concentration of LiF, which is inherently an insulating material, surpasses a critical threshold, a significant decline in capacity is observed.

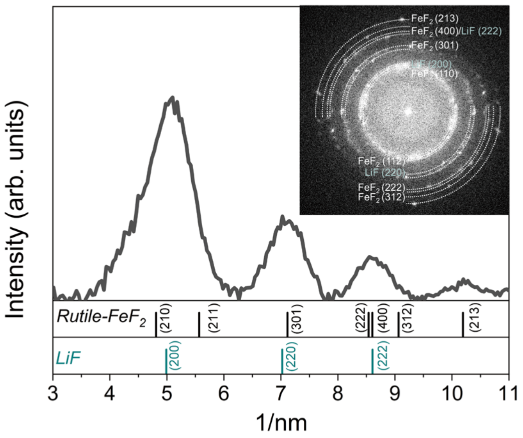

Supplementary Figure S2: FFT Analysis of LiF-FeF_2 Nanocomposite. Azimuthal integration of FFT pattern (Fig. 1b) for LiF-FeF_2 nanocomposite.

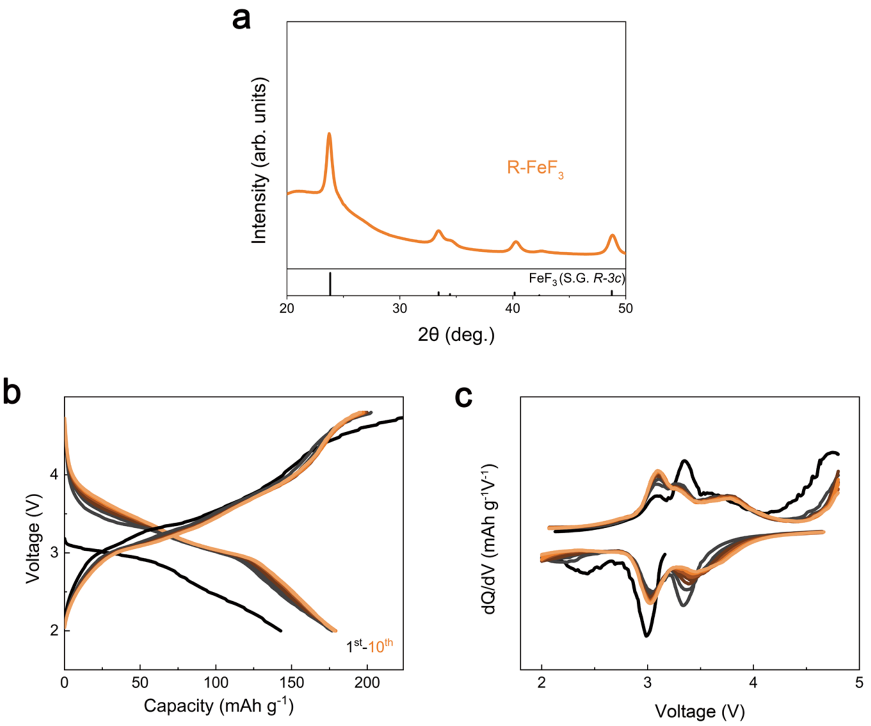

Supplementary Figure S3: Characterization of Rhombohedral FeF_3. (a) Powder X-ray diffraction patterns of the rhombohedral FeF_3 (R-FeF_3). **(b)** Electrochemical profile of R-FeF_3 at 25 °C and current density of 20 mA g^{-1}. **(c)** Differential analysis of the voltage profile.

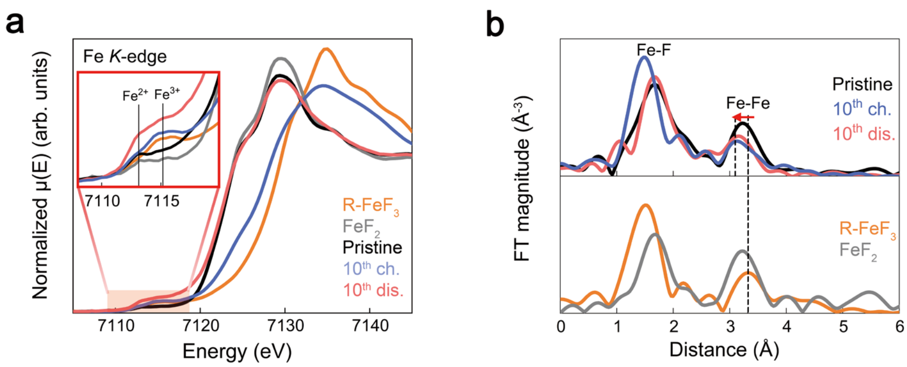

Supplementary Figure S4: Fe K-edge XAS Analysis of Pristine and Cycled States. (a, b) Ex situ XANES and EXAFS spectra of the Fe *K*-edge for pristine and 10th charged/discharged state. The inset shows an expanded view of the pre-edge region.

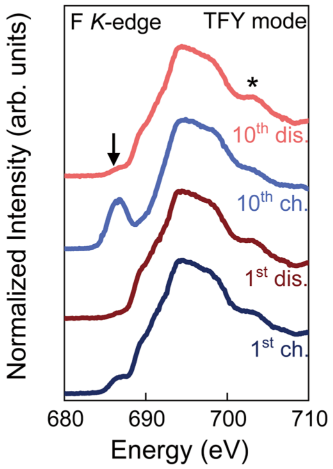

Supplementary Figure S5: F K-edge XAS Analysis During Cycling. F *K*-edge XAS spectra of the LiF-FeF_2 in total fluorescence yield (TFY) at 1st and 10th cycles. The asterisk is the peak corresponding to LiF.

Supplementary Note 2. Origin of Fluoride Ions in the Formation of T-FeF_3

To investigate the origin of fluoride ions (F^{-}) in the formation of tetragonal FeF_3 (T-FeF_3), F K-edge spectra analysis was conducted (Supplementary Figure S5). The F *K*-edge spectra in total fluorescence yield (TFY) mode for the 1st and 10th cycles show a distinct pre-edge peak around 684.0 eV that becomes more prominent upon cycling. This peak, commonly observed in highly oxidized iron fluorides and oxyfluorides, relates to the transition of F 1s to 2p orbitals hybridized with Fe 3d orbitals[1–5], suggesting the formation of $Fe^{3+}F_3$. For $Fe^{3+}F_3$ to form electrochemically, F ions from LiF must act as charge neutralizers during charging process. Since the electrochemical measurements were conducted in the presence of F ion-containing binders and electrolytes (Fig. 1c), we confirmed the electrochemical profile in the absence environment of other F ion sources except LiF (Supplementary Figure S6). The evolution of the 4 V plateau was observed without significant changes in the electrochemical profile. This suggests that the reaction between LiF and FeF_2 forms an electrochemically active FeF_3 with a tetragonal-like polymorph.

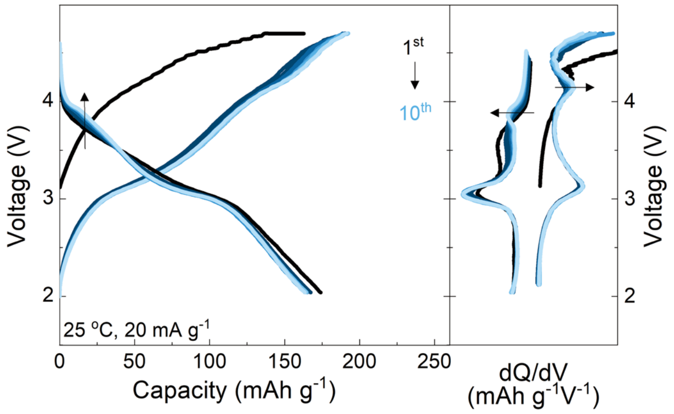

Supplementary Figure. S6: Electrochemical Behavior of LiF-FeF$_2$. Without External Fluorine Sources. Electrochemical profile of LiF-FeF$_2$ nanocomposite without additional fluorine sources other than LiF, measured at 25 °C and a current density of 20 mA g^{-1}.

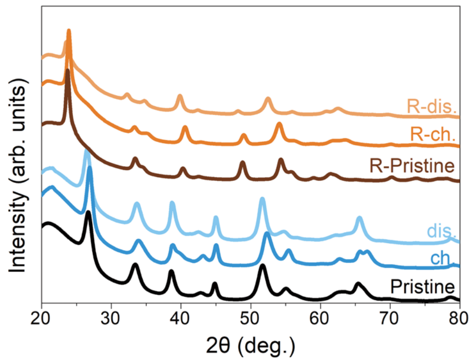

Supplementary Figure S7: Structural Comparison of LiF-FeF_2 and R-FeF_3 During Cycling. Ex situ XRD patterns of LiF-FeF_2 and R-FeF_3 electrodes at pristine and 10th charged/discharged states.

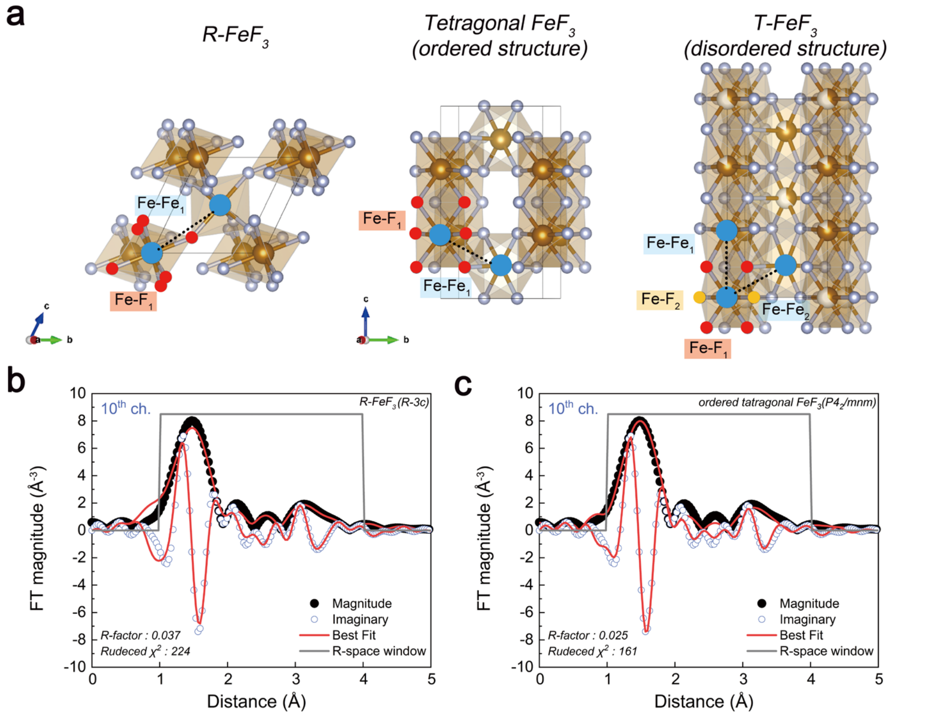

Supplementary Figure S8: Comparison of FeF_3 Polymorph Structures *via* EXAFS Fits. **(a)** Crystal structures of ordered tetragonal FeF_3, T-FeF_3 (disordered structure), and R-FeF_3. Brown and silver balls indicate Fe and F ions, respectively. Fourier transformed magnitude (black), imaginary part (blue), and best fit (red) using **(b)** the R-FeF_3 model and **(c)** the ordered tetragonal FeF_3 model for the 10th charged state electrode**.** (See Supplementary Table S2.)

Supplementary Note 3. Identification of Electrochemically Induced Polymorphs in the Local Environment

The tetragonal structure of FeF_3 derived from $Li_{0.5}FeF_3$ has been reported in two forms based on the ordering of Fe: the ordered tetragonal FeF_3, where Fe is occupied at the 4e site, and the disordered tetragonal FeF_3, where Fe is randomly distributed at the 2a and 4e sites[6]. The arrangement of Fe affects the local environment of Fe and, consequently, the EXAFS spectrum[7]. In the EXAFS spectrum of the 10th charged state of LiF-FeF_2 shown in Supplementary Figure S4b, a shorter Fe-Fe distance is observed compared to R-FeF_3. Both ordered tetragonal FeF_3 and R-FeF_3 exhibit a Fe-Fe distance of ~3.7 Å, while disordered tetragonal FeF_3 shows Fe-Fe distances of 3.16 Å and 3.69 Å. These differences can be attributed to structural variations among ordered tetragonal FeF_3, disordered tetragonal FeF_3, and R-FeF_3. To elucidate the structure induced by LiF-FeF_2, we performed EXAFS fitting for each structure (Supplementary Figure S8a). The disordered tetragonal FeF_3 (Fig. 1f) exhibited significantly lower reduced χ^2 and R-factor values compared to R-FeF_3 and ordered tetragonal FeF_3 (Supplementary Figures S8b and S8c), indicating that the local environment is more consistent with the disordered tetragonal structure.

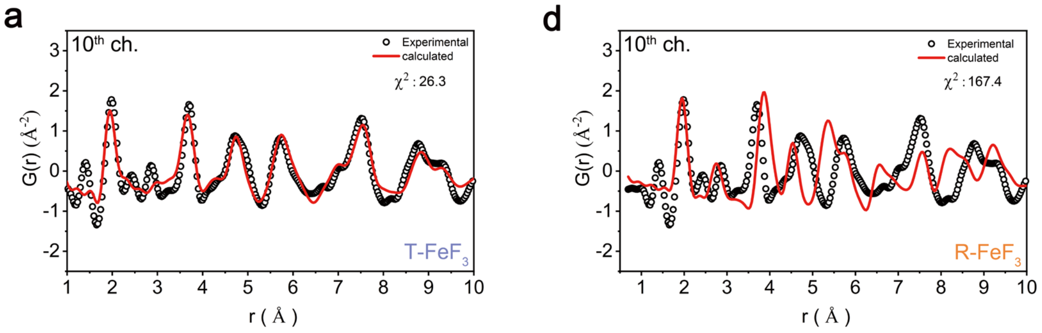

Supplementary Figure S9: Atomic pair distribution function (PDF) Analysis of FeF_3 Polymorphs at 10th Charge. Pair distribution function (PDF) fit for **(a)** T-FeF_3 and **(b)** R-FeF_3 structures in the 10th charged state.

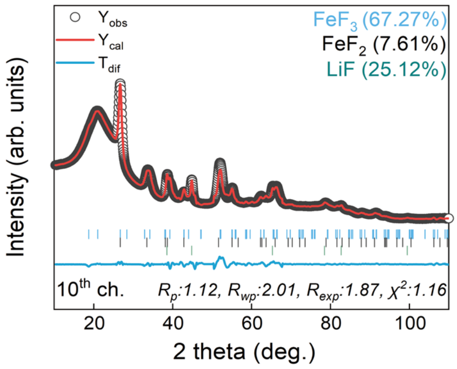

Supplementary Figure S10: XRD Refinement of 10th Charged Electrode. Rietveld refinement of the XRD data (λ = 1.5406 Å) of the 10th charged state. (See Supplementary Table S3.)

Supplementary Note 4. Identification of a Tetragonal Polymorph Electrochemically Derived from LiF-FeF_2

Previous studies have reported the electrochemical induction of a tetragonal FeF_3 structure from $Li_{0.5}FeF_3$[6]. The reported tetragonal FeF_3 shares the same anion framework as FeF_2 (*P4₂/mnm*) but differs in the occupancy of Fe sites. Based on the similarity between the XRD pattern of the 10th charged state (Supplementary Figure S7) and that of FeF_2, along with the characteristic 4 V plateau observed in the charge/discharge profile (Fig. 1c), we identified tetragonal FeF_3 as a potential polymorph electrochemically induced from LiF-FeF_2. Rietveld refinement of the XRD pattern at the 10th charged state, where the 4 V plateau is prominently formed, confirmed a good fit with the tetragonal FeF_3 structure (Supplementary Figure S10).

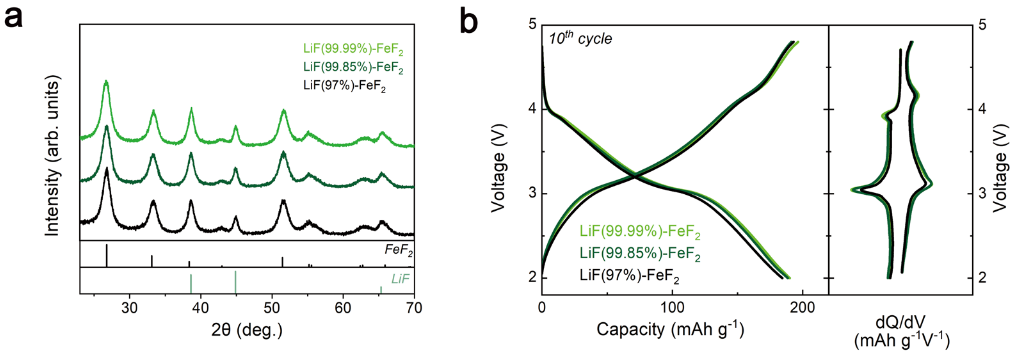

Supplementary Figure S11: Impact of LiF Purity on LiF-FeF_2 Nanocomposites. (a) XRD patterns of LiF-FeF_2 nanocomposites prepared using LiF of different purities. (b) Electrochemical profile and differential analysis of voltage profile at 10th cycle, measured at 25 °C and a current density of 20 mA g^{-1}.

Supplementary Note 5. Role of LiF-FeF_2 Interfacial Integrity in Phase Evolution and Electrochemical Performance of T-FeF_3.

The interfacial contact between LiF and FeF_2 in the LiF-FeF_2 nanocomposite plays a critical role in facilitating the guided phase transition toward T-FeF_3 and determining the overall electrochemical performance. To systematically investigate the impact of this interfacial contact, we examined two key factors: the purity of the LiF precursor, which influences the chemical integrity of the composite, and the ball-milling time, which affects particle size and mixing uniformity.

The critical role of LiF-FeF_2 interfacial contact in forming T-FeF_3 was further supported by investigating the effect of LiF precursor purity. To evaluate this, we prepared LiF-FeF_2 composites using LiF with purities of 99.99%, 99.85%, and 97%. XRD analysis revealed no significant differences in crystal structure or phase composition among the samples, indicating that variations in purity did not cause noticeable changes in the bulk structure (Supplementary Figure S11a). However, electrochemical measurements revealed that lowering the LiF purity led to a slight but consistent reduction in capacity. This capacity loss is likely due to the presence of impurities, which may disrupt the LiF-FeF_2 interface, reduce effective contact, or introduce electrochemically inactive phases that interfere with smooth T-FeF_3 formation (Supplementary Figure S11b).

To further confirm the importance of interfacial contact, we investigated the effect of crystallinity by systematically varying the ball-milling time. By adjusting only the initial LiF-FeF_2 mixing duration (48 h, 12 h, and 0 h) while maintaining the carbon mixing step constant at 12 h (500 rpm), we were able to isolate the impact of interfacial contact and crystallinity on electrochemical behavior. XRD analysis revealed that reducing the milling time resulted in narrower diffraction peaks and a noticeable decrease in the full width at half maximum (FWHM), indicating increased crystallinity and larger particle sizes

(Supplementary Figure S12a and b). This microstructural change reduced the interfacial contact area between LiF and FeF_2, which is essential for promoting the phase transition to T-FeF_3. Electrochemical testing further confirmed that insufficient LiF-FeF_2 contact deteriorates capacity but has limited impact on voltage hysteresis behavior. As milling time decreased, charge–discharge profiles (Supplementary Figure S11c) showed reduced capacity due to poorer interfacial contact. This capacity degradation is attributed to the limited formation of electrochemically active T-FeF_3, resulting in compositional inhomogeneity. However, GITT (Galvanostatic Intermittent Titration Technique) analysis revealed that the difference in relaxed voltages between charge and discharge (V_{gap}), representing reaction pathway-dependent kinetic hysteresis arising from phase-transition and bond-breaking barriers, remained nearly constant across all milling times and significantly smaller than that of R-FeF_3 (Supplementary Figures S12d–e). The rest potential change (ΔV_{rest}), which reflects kinetic polarization, slightly increased at the end of charge as milling time decreased. Nevertheless, even in the shortest milling condition (0 h), ΔV_{rest} was still considerably smaller than in R-FeF_3 (Supplementary Figure S12f), indicating that kinetic effects such as electron/ion diffusion barriers were minimal. This is likely because our nanocomposite system consists of sufficiently nanosized particles and a highly conductive carbon matrix, minimizing the impact of transport-related kinetic limitations.

In summary, the interfacial contact between LiF and FeF_2 plays a crucial role in the formation and electrochemical activation of T-FeF_3. In our nanocomposite system consisting of sufficiently small particles and an abundant conductive carbon matrix, changes in interfacial contact significantly affect capacity but have minimal impact on voltage hysteresis. Notably, T-FeF_3 exhibits consistently low hysteresis even under conditions of degraded interfacial contact, reinforcing the conclusion that the hysteresis difference between T-FeF_3 and R-FeF_3 arises primarily from their inherent reaction-pathway-governed kinetic behavior, associated with differences in phase transition reversibility and bond-breaking energetics, rather than from kinetic limitations such as electron or ion transport resistance.

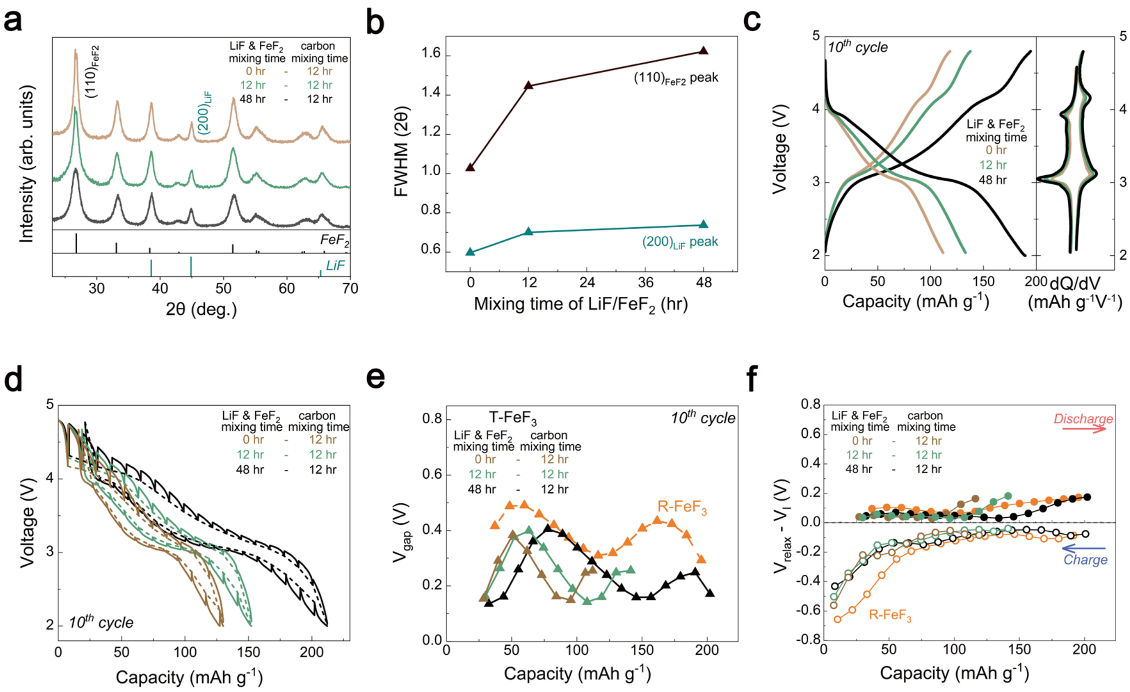

Supplementary Figure S12: Influence of Mixing Time on Structural and Electrochemical Properties. (a) XRD patterns of LiF-FeF_2 nanocomposites prepared with different mixing times. **(b)** Full width at half maximum (FWHM) of the $(110)_{FeF2}$ and $(200)_{LiF}$ diffraction peaks as a function of mixing time. **(c)** Electrochemical charge-discharge profiles of the composites prepared with varying mixing times, along with their differential voltage (dQ/dV) analysis shown on the right. **(d)** GITT profiles at the 10th cycle. The cells were allowed to relax for 3 h after every 11.2 mAh g^{-1} (corresponding to 0.05 e^- per formula unit) of discharge/charge at a current density of 20 mA g^{-1} at 25 °C. **(e)** Voltage gap ($V_{gap} = V_{relax,charge} - V_{relax,discharge}$) measured after 3 h relaxation at equivalent lithiation states for each mixing condition. **(f)** Voltage changes after 3 h relaxation (ΔV_{rest}) measured at different lithiation states during discharge and charge for each mixing condition.

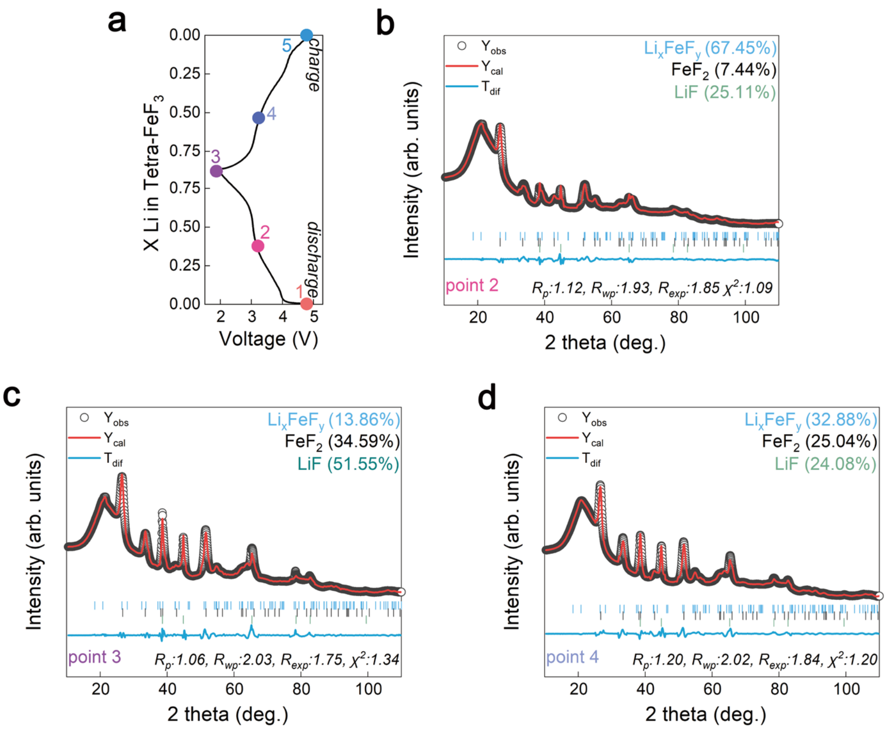

Supplementary Figure S13: Rietveld Analysis of T-FeF$_3$ at Various Lithiated States. (a) Voltage-time profile of T-FeF$_3$ measured at 25°C and 20 mA g^{-1}. Rietveld refinement of the XRD data (λ = 1.5406 Å) of **(b)** half-discharged state (point 2), **(c)** discharged state (point 3), and **(d)** half-charged state (point 4). (See Supplementary Table S4.)

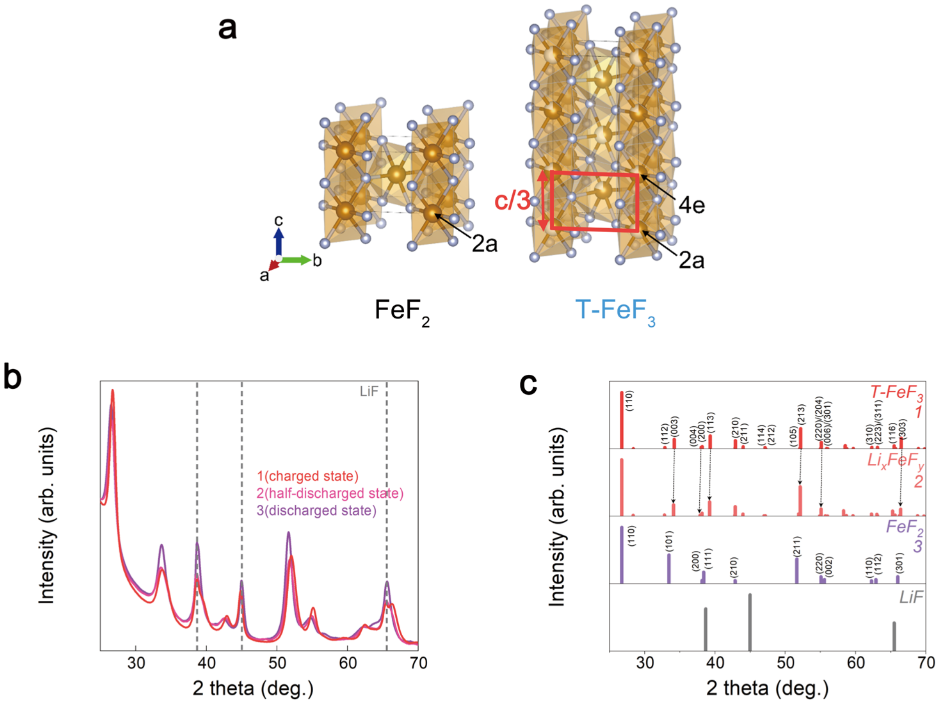

Supplementary Figure S14: Structural Evolution of T-FeF_3 During Cycle. (a) Crystal structure of T-FeF_3 and FeF_2. Since T-FeF_3 is similar to a three-fold stacking of the FeF_2 anion framework. Brown and silver balls indicate Fe and F ions, respectively. **(b)** Ex situ XRD patterns of T-FeF3 for (1) charge to 4.8 V, (2) half-discharge to 3.4 V, and (3) discharge to 2 V. **(c)** XRD patterns (λ = 1.5406 Å) for the main *$P4_2/mnm$* structure with the largest phase fraction at each state. (See Fig. 2a-c.)

Supplementary Note 6. XRD Pattern Changes During Discharge of T-FeF_3.

Although T-FeF_3 and FeF_2 share the same space group (*$P4_2/mnm$*), their unit cell parameters differ, leading to distinct diffraction patterns for T-FeF_3 compared to FeF_2 (Supplementary Figures S14a and 14b). These peaks become increasingly pronounced as T-FeF_3 is electrochemically formed during the initial cycles, as shown in Fig. 1d. During discharge, a gradual shift of the XRD peaks toward lower angles is observed when comparing patterns of point 1 (charged state) and point 2 (half-discharged state) in Supplementary Figure S14c. Specifically, shifts in the (003), (113), (213), and (303) peaks of T-FeF_3, corresponding to 34.8°, 40°, 52°, and 66.7°, are clearly identifiable. Among these, the (003), (113), and (303) peaks shift to lower angles, leading to peak broadening due to overlap with FeF_2 or LiF peaks present in the same state. At point 3 (discharged state), only FeF_2 and LiF peaks are sharply observed (Supplementary Figure S14b).

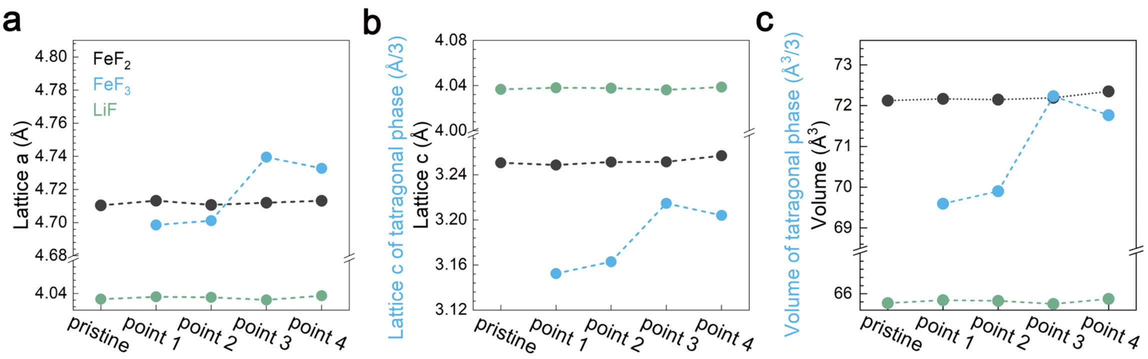

Supplementary Figure S15: Lattice Parameter and Volume Changes of T-FeF_3 During Cycling. Comparison of refined crystallographic parameters at each voltage. **(a)** Lattice a parameter, **(b)** lattice c parameter, and **(c)** volume. The c lattice parameter and volume of T-FeF_3 were expressed as Å/3 and Å^3/3 to compare with FeF_2. (See Supplementary Tables S3 and S4.)

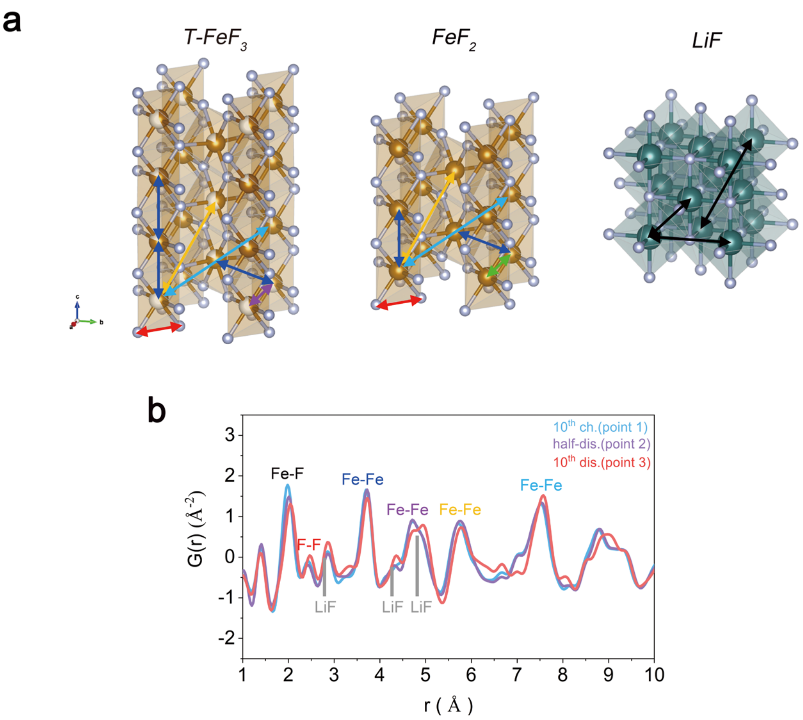

Supplementary Figure S16: PDF Analysis of T-FeF_3 During Charge and Discharge. (a) Crystal structures of T-FeF_3, FeF_2, and LiF. Each arrow corresponds to each peak in the PDF. The brown, silver, and green balls represent Fe, F, and LiF ions, respectively. **(b)** PDF comparison for 10th charge (point 1), half-discharge (point 2), and 10th discharge (point 3).

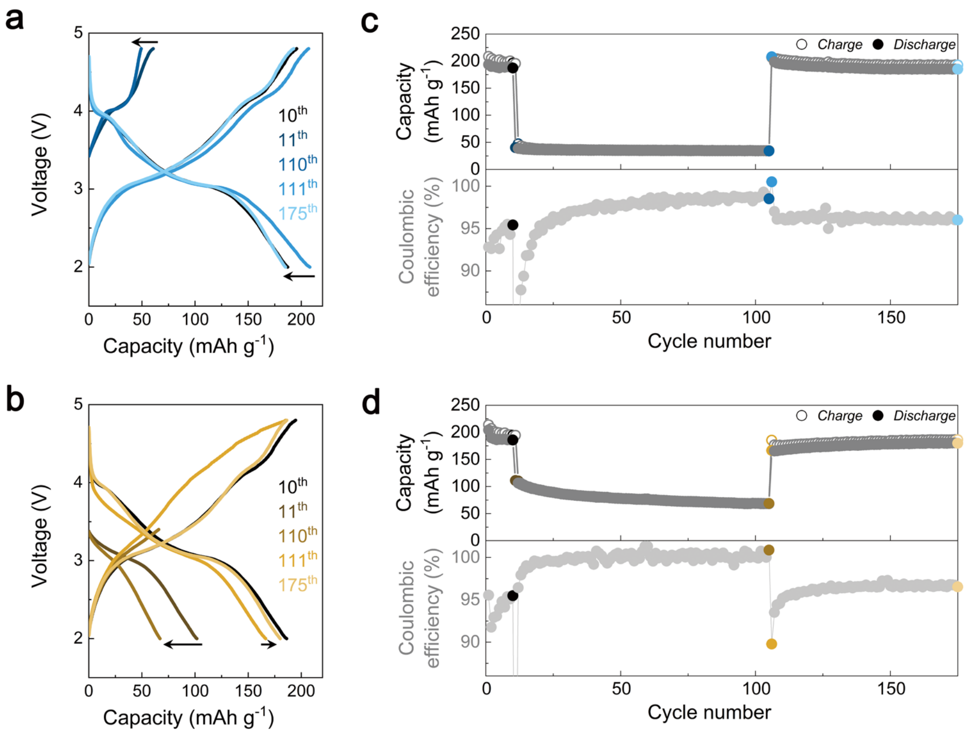

Supplementary Figure S17: Electrochemical Reversibility of T-FeF_3 Under Different Voltage Ranges. Voltage profile and cycle performance of T-FeF_3 in the order of **(a, c)** WV-UV-WV range and **(b, d)** WV-LV-WV range, measured at 25 °C and a current density of 20 mA g^{-1}. Arrows have been added to indicate the progression of voltage profiles across different cycles.

Supplementary Note 7. Evaluation of the Reversibility of T-FeF_3

Supplementary Figure S14 demonstrates the reversibility across different voltage ranges after the initial 10 cycles required to form T-FeF_3. At the 100th cycle, the wide voltage (WV, Supplementary Figure S1c) and upper voltage ranges (UV) show excellent capacity retention of 99 % and 93 %, respectively, while the lower voltage range (LV) exhibits relatively poor capacity retention of 69 %. The excellent reversibility in the UV and the rapid capacity decay in the LV suggest that the reaction mechanism of T-FeF_3 proceeds through Li^+ insertion and conversion reaction.

During the charging process, the phase fraction changes gradually through points 4 and 5, unlike the stepwise changes observed during discharge (Fig. 2c). The formation of T-FeF_3 from LiF and FeF_2 requires the splitting of LiF:

$$LiF + FeF_2 \rightarrow FeF_3 + Li^+$$

LiF splitting requires a high-voltage environment[8–12]. Insufficient LiF splitting in the LV may lead to incomplete formation of T-FeF_3 and accumulation of inactive LiF, resulting in capacity decay. To verify insufficient LiF splitting in the LV, reversibility was checked in the UV and LV, followed by a cycle in the WV (Supplementary Figure S17). The electrochemical profile and capacity in UV remained almost identical to the 10th cycle when re-measured in the WV. In contrast, for the 111th voltage profile in the LV region, a large overpotential was observed when the charged cut-off voltage was restored from 3.4 V to 4.8 V. This behavior, coupled with the absence of the 4 V plateau and reduced capacity, suggests a failure to form T-FeF_3 due to insufficient LiF splitting. However, as cycling continued in the WV range, the gradual evolution of the 4 V plateau and the concurrent increase in capacity clearly indicate successful T-FeF_3 formation under high-voltage operation. These characteristics of electrochemical behavior across various voltage ranges provide further evidence that T-FeF_3 undergoes both insertion and conversion reaction mechanisms.

Furthermore, this result highlights the necessity of a higher charge cut-off voltage for the effective formation of T-FeF_3. This is supported by a comparative analysis of electrochemical behavior at 4.8 V and 4.5 V. As shown in Supplementary Fig. S18a, the sample cycled at 4.8 V exhibited a well-defined plateau and significantly higher capacity, while the sample cycled at 4.5 V showed a much less pronounced 4 V feature, suggesting incomplete LiF splitting and limited activation of T-FeF_3. In contrast, R-FeF_3 exhibited little difference in capacity depending on the charge cut-off voltage, but consistently showed lower capacity retention compared to T-FeF_3, regardless of the voltage range (Supplementary Figures S18b and c). This further emphasizes the structural irreversibility of R-FeF_3 and its inferior electrochemical reversibility. These findings collectively reinforce that 4.5 V is insufficient to fully promote the reconversion reaction, and that a 4.8 V cut-off is essential to separating LiF required for reversible T-FeF_3 formation.

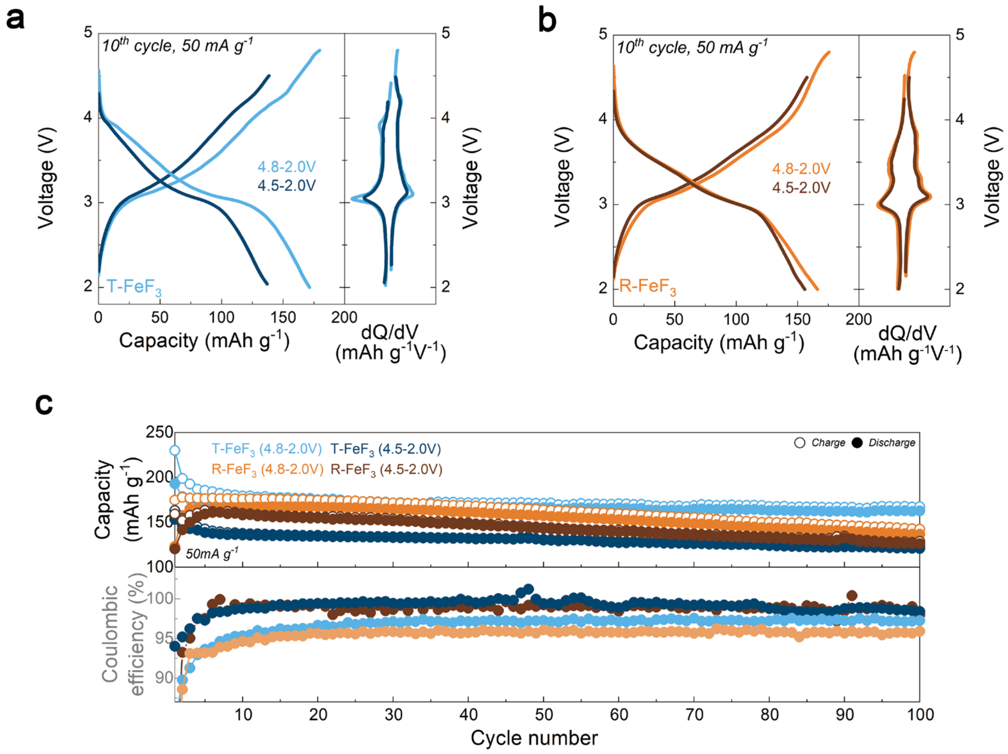

Supplementary Figure S18: Effect of Charge Cut-Off Voltage on Electrochemical Performance of T-FeF_3 and R-FeF_3. (a, b) Electrochemical profiles of T-FeF_3 and R-FeF_3 at the 10th cycle measured at 50 mA g^{-1} under different charge cut-off voltages (4.5 V and 4.8 V) at 25 °C. **(c)** Cycling stability and columbic efficiency of T-FeF_3 and R-FeF_3 at 50 mA g^{-1} under different charge cut-off voltages (4.5 V and 4.8 V) at 25 °C.

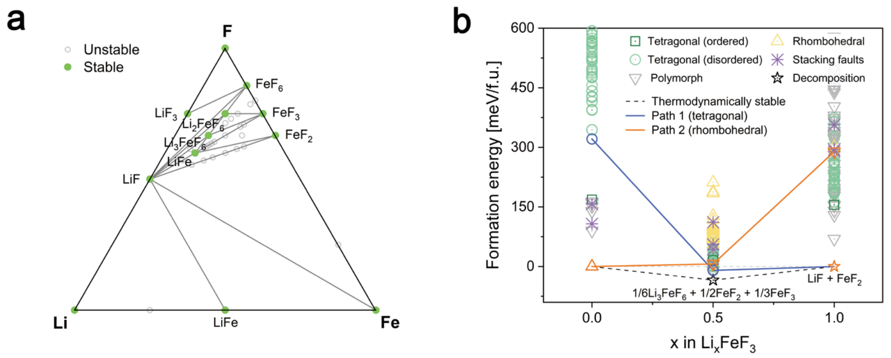

Supplementary Figure S19: Phase Stability and Reaction Pathways in the Li–Fe–F System. (a) Phase diagram of Li-Fe-F system. **(b)** The formation energies as a function of Li contents with all considered structures (ordered and disordered tetragonal, rhombohedral, stacking faulted[13], various polymorphs of each composition from materials project[14]. The dashed line is the convex hull of the formation energies, and the red and navy solid lines are the suggested reaction path according to the host structure (tetragonal and rhombohedral structure).

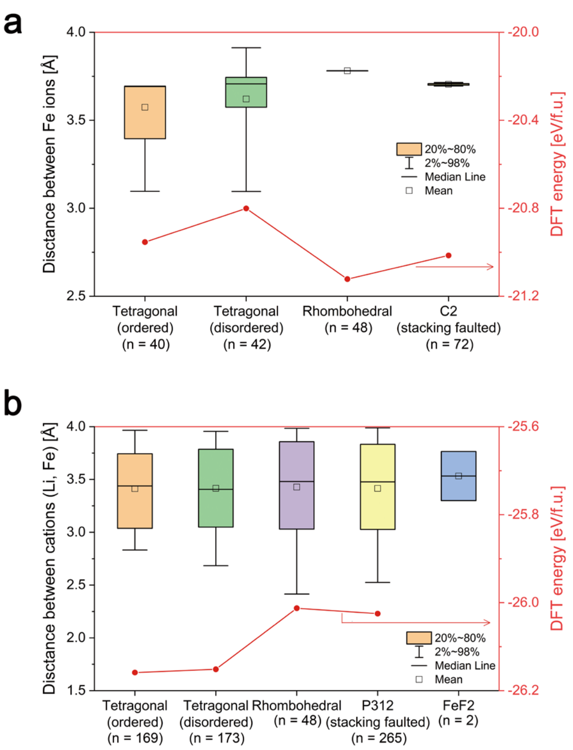

Supplementary Figure S20: Structural and Energetic Comparison at The Fully Delithiated State and The Fully Lithiated State. The distance between cations (Li and Fe) and calculated energy of each structure (a) the fully delithiated state (x=0), (b) the fully lithiated state (x=1). n in x label represents the number of distance data between cations. (See Supplementary Tables S5 and S6.)

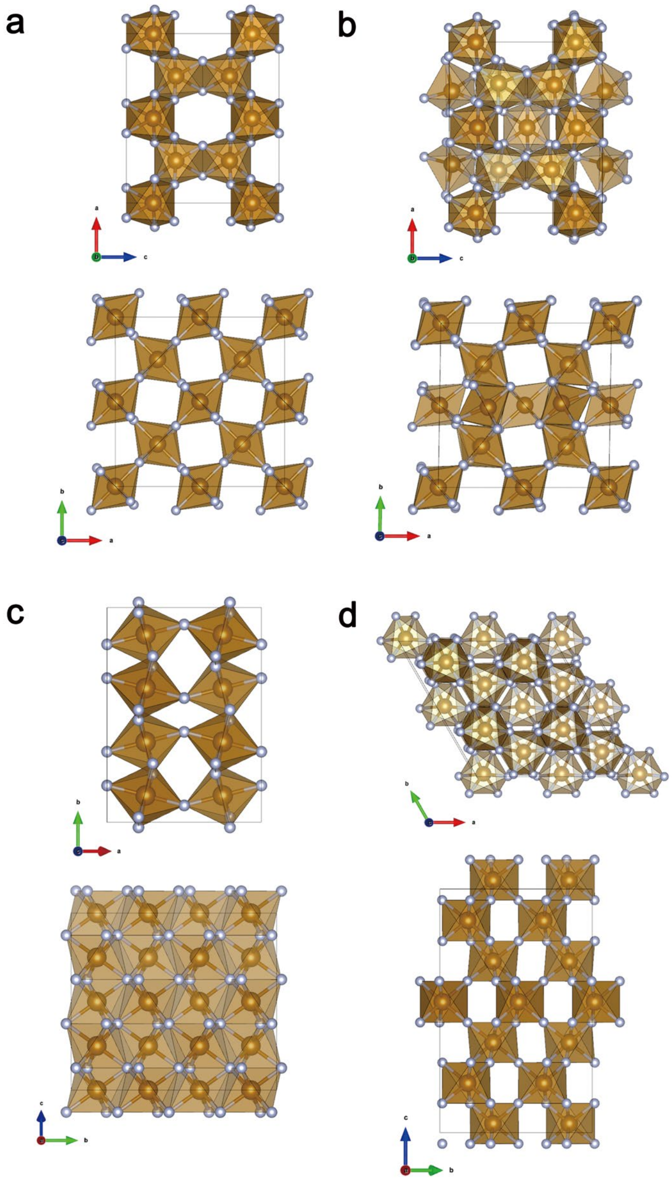

Supplementary Figure S21: The structures of FeF_3 polymorphs. (a) Ordered tetragonal, **(b)** disordered tetragonal, **(c)** rhombohedral, and **(d)** stacking faulted (P312) structure. Fe, F are in gold, and silver balls, respectively.

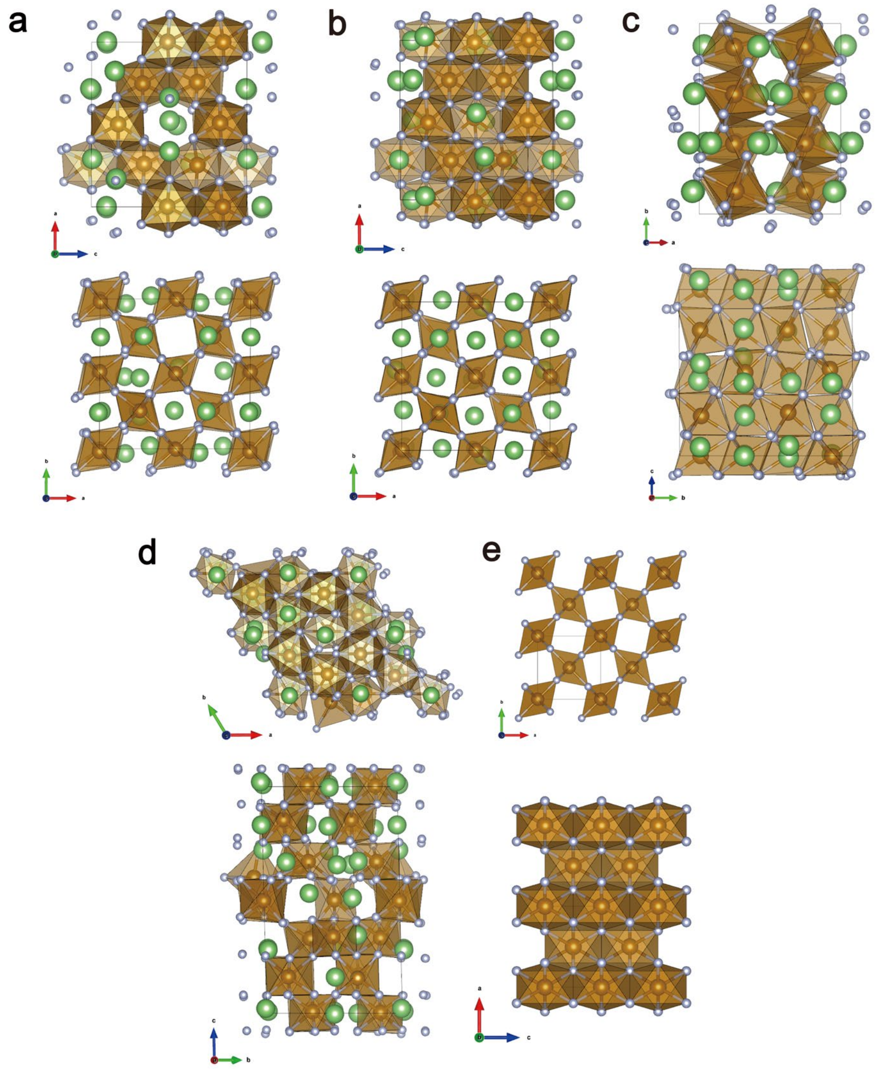

Supplementary Figure S22: The Structures of $LiFeF_3$ Polymorphs. **(a)** Ordered tetragonal, **(b)** disordered tetragonal, **(c)** rhombohedral, **(d)** stacking faulted (P312) structures. **(e)** FeF_2 is one of decomposition products of $LiFeF_3$. Li, Fe, F are in green, gold, and silver balls, respectively.

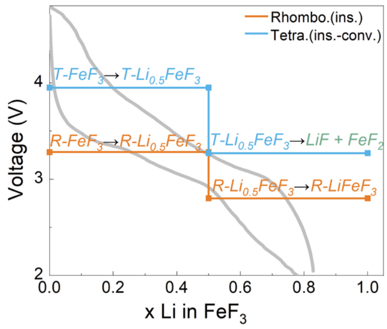

Supplementary Figure S23: Voltage Profile of R-FeF_3 Based on Experiment and DFT. Experimentally measured voltage profile and DFT calculated reaction voltage for R-FeF_3 at different states of lithiation.

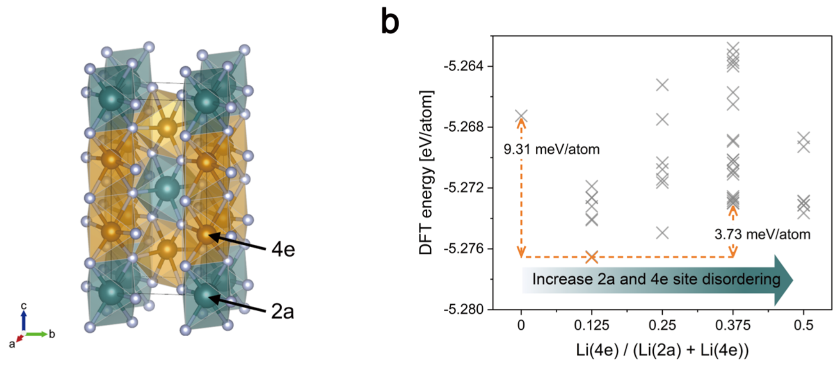

Supplementary Figure S24: Site Preference and Energetics of Li and Fe in Tetragonal $Li_{0.5}FeF_3$. The Li and Fe site preference between 2a and 4e sites in tetragonal $Li_{0.5}FeF_3$ structure. **(a)** The crystal structure of ordered tetragonal $Li_{0.5}FeF_3$ ($P4_2/mnm$). Green, brown, and silver balls indicate Li, Fe, and F ions. **(b)** The DFT energy of tetragonal $Li_{0.5}FeF_3$ as a function of the fraction of Li occupancy in the 4e site. The red mark indicates the lowest energy among Li and Fe site-disordered structures.

Supplementary Note 8. Li and Fe Site Disordering in Tetragonal $Li_{0.5}FeF_3$

In the ordered tetragonal $Li_{0.5}FeF_3$ structure ($P4_2/mnm$), Li and Fe atoms are located at 2a and 4e sites, respectively. Our experimental results, along with previous reports[6], indicate that site disordering between Li and Fe atoms occurs during cycling (Supplementary Note 3 and Figure S10). To investigate the stability of cation disordering, we generated Li and Fe site-disordered structures using enumeration techniques and obtained their energies through DFT calculations (Supplementary Figure S24). The most stable configuration is a slightly disordered structure where 0.125 Li atoms occupy the 4e site and the rest occupy the 2a site. The energy difference between this most stable and ordered configurations is 9.31 meV/atom. Note that the Li and Fe site-disordered structure based on the previous report[6] is 3.73 meV/atom higher than our most stable configuration. Although this configuration is less stable than the most stable structure, their energy difference is quite small, thus, they can coexist due to entropy at room temperature.

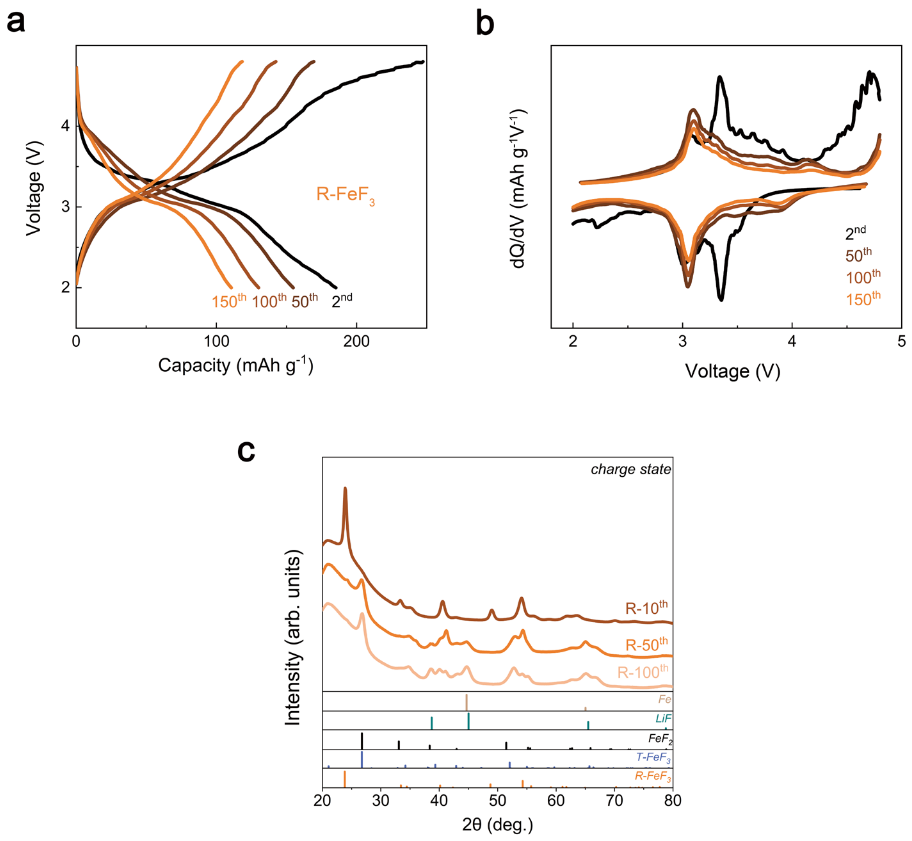

Supplementary Figure S25: Long-Term Electrochemical Behavior and Structural Evolution of R-FeF_3. (a,b) Electrochemical profile and differential analysis of voltage profile according to long-term cycling of R-FeF_3 at 25 °C and 20 mA g^{-1} current density. (c) ex situ XRD patterns for the 10th, 50th, and 100th charge states.

Supplementary Note 9. Irreversible Phase Transition of R-FeF_3 During Long-Term Cycling

Recent studies on the reaction mechanism of R-FeF_3 have reported that Li insertion into R-FeF_3 forms $Li_xFe_yF_3$, a structure deficient in Fe, leading to the irreversible phase displacement of FeF_2 into a tetragonal structure from the first discharge cycle[13]. Initially, the crystallinity of the displaced FeF_2 is low and is only detectable through PDF analysis. However, with repeated charge/discharge cycles, the accumulation of irreversible FeF_2 may become observed in XRD patterns. Long-term cycling experiments were performed to investigate the irreversible formation of FeF_2 (rcp-tcp transition) in the reaction mechanism of R-FeF_3.

As shown in Supplementary Figure S25a, a gradual decrease in capacity is observed as cycling progresses. Interestingly, a slight 4 V plateau characteristic of T-FeF_3 emerges with continued cycling. If this electrochemical feature is due to the irreversible rcp-tcp transition, FeF_2 should be observable in the charged state. To confirm this, we examined ex situ XRD patterns of the 50th and 100th charge states, where the 4 V redox peak starts to appear in dQ/dV

and becomes more pronounced, respectively (Supplementary Figures S25c and S25b). Up to the 10th cycle, the XRD pattern primarily exhibits rhombohedral structure peaks. However, as cycling progresses, the main peak of the rhombohedral structure at 23.9° gradually diminishes. Conversely, the peak at 26.8° corresponding to the tetragonal structure (indicated by black arrows) becomes more prominent. Additionally, peaks corresponding to LiF and Fe metal (at 44.7°, indicated by green arrows) also emerge with continued cycling. These observations strongly support the inclusion of an irreversible rcp-tcp transition in the reaction pathway of R-FeF_3.

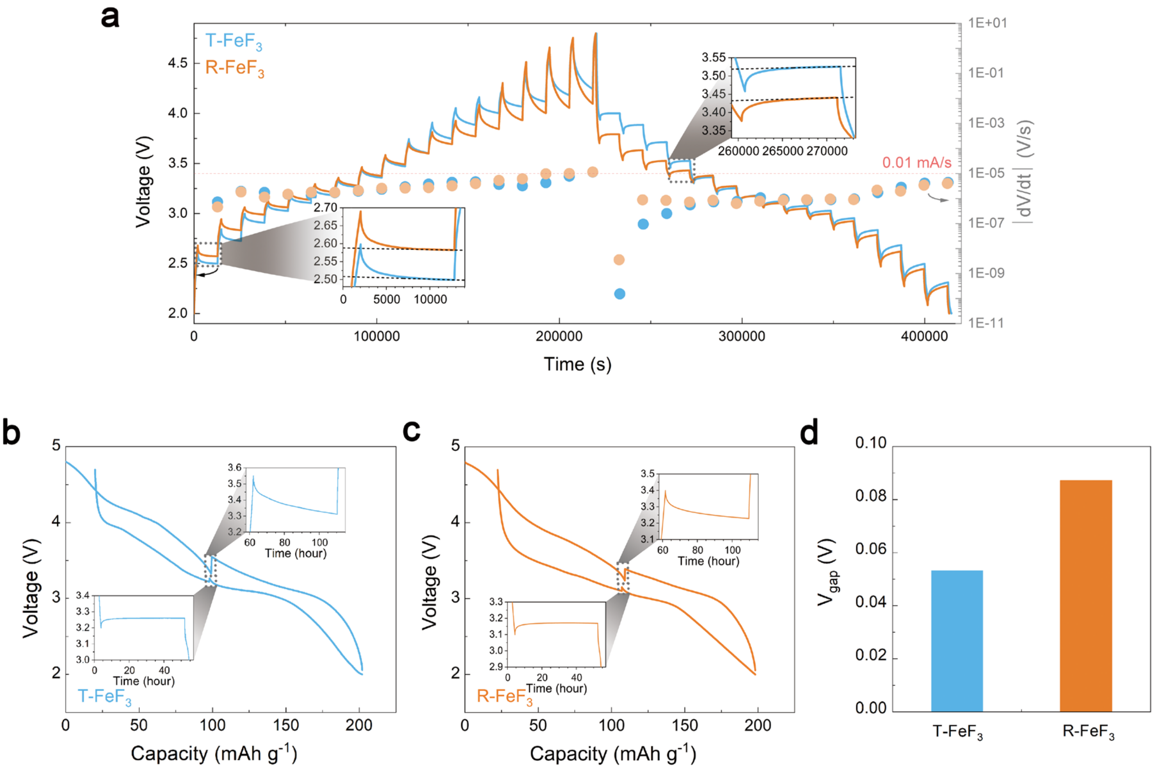

Supplementary Figure S26: Effect of Long-Term Relaxation on Voltage Hysteresis. (a) GITT profiles of T-FeF_3 and R-FeF_3 after the 10th cycle, along with the absolute value of the voltage change rate (dV/dt) in each relaxation region. Cells were allowed to relax for 3 h after every 11.2 mAh g^{-1} (corresponding to 0.05 e^- per formula unit) of charge or discharge at a current density of 20 mA g^{-1} at 25 °C. The time-dependent voltage profile is shown in the inset, with the voltage variation indicated by the dotted line. Long-term (48 h) relaxation tests where voltage hysteresis is similar after the 10th cycle for **(b)** T-FeF_3 and **(c)** R-FeF_3 measured at 25°C and a current density of 20 mA g^{-1}. **(d)** Voltage difference ($V_{gap} = V_{relax, charge} - V_{relax, discharge}$) between charge and discharge steps after 48 h relaxation.

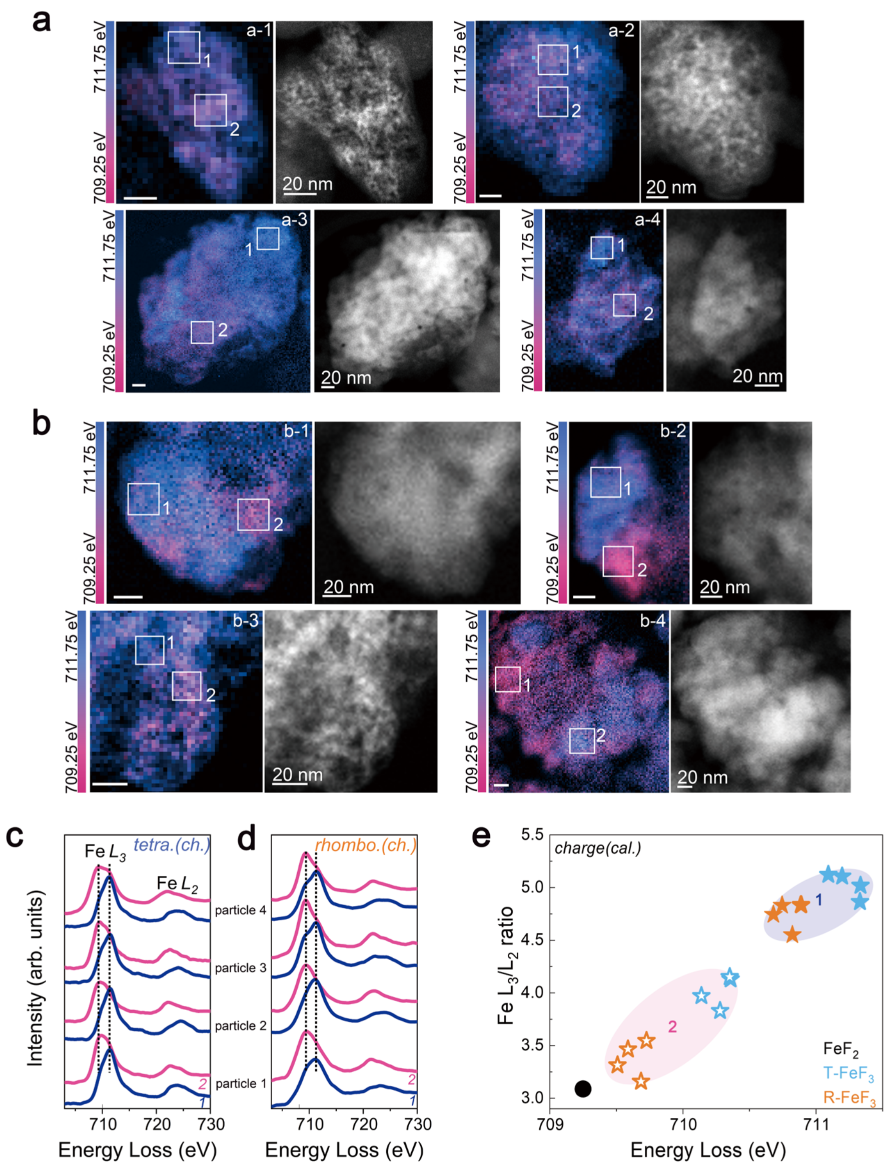

Supplementary Figure S27: Fe Oxidation State Distribution Among Various Particles at the 10th Charged State. (a,b) STEM-EELS images of T-FeF_3 and R-FeF_3 at 10th charged state for the energy distribution of the Fe L_3-edge peak. These measurements were taken at a current density of 20 mA g^{-1} at 25°C. The scale bar in all images represents 20 nm. Fe $L_{3,2}$-edge spectra of **(c)** T-FeF_3 and **(d)** R-FeF_3 at the 10th charge state for particles 1-4. 1(blue) and 2(pink) correspond to the most oxidized and most reduced regions, respectively. **(e)** Fe L_3-edge peak positions and L_3/L_2 intensity ratios for different particles (n = 4) at each TC and RC. Closed and hollow symbols indicate the most oxidized and most reduced regions at each particle, respectively.

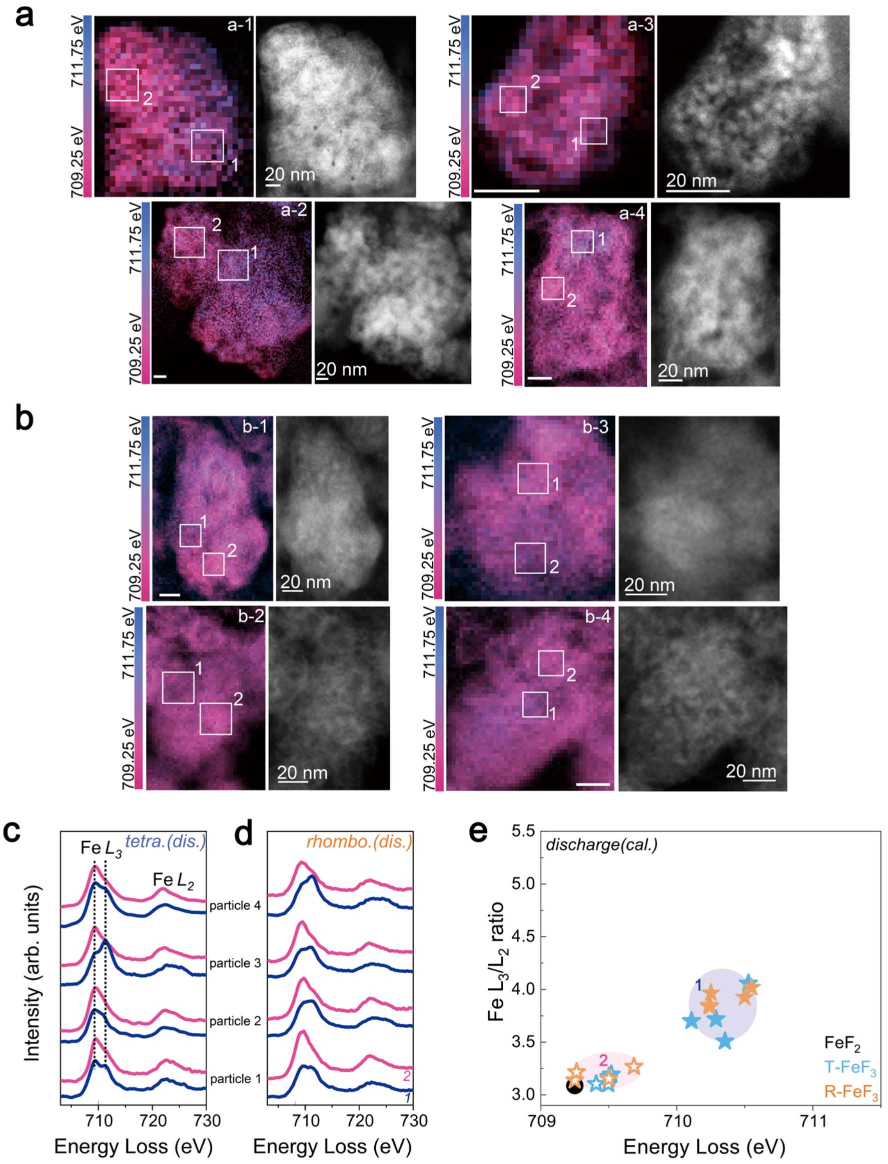

Supplementary Figure S28: Fe Oxidation State Distribution Among Various Particles at the 10th Discharged State. (a,b) STEM-EELS images of T-FeF_3 and R-FeF_3 at 10th discharged state for the energy distribution of the Fe L_3-edge peak. These measurements were taken at a current density of 20 mA g^{-1} at 25°C. The scale bar in all images represents 20 nm. Fe $L_{3,2}$-edge spectra of **(c)** T-FeF_3 and **(d)** R-FeF_3 at the 10th discharge state for particles 1-4. 1(blue) and 2(pink) correspond to the most oxidized and most reduced regions, respectively. **(e)** Fe L_3-edge peak positions and L_3/L_2 intensity ratios for different particles (n = 4) at each TD and RD. Closed and hollow symbols indicate the most oxidized and most reduced regions at each particle, respectively.

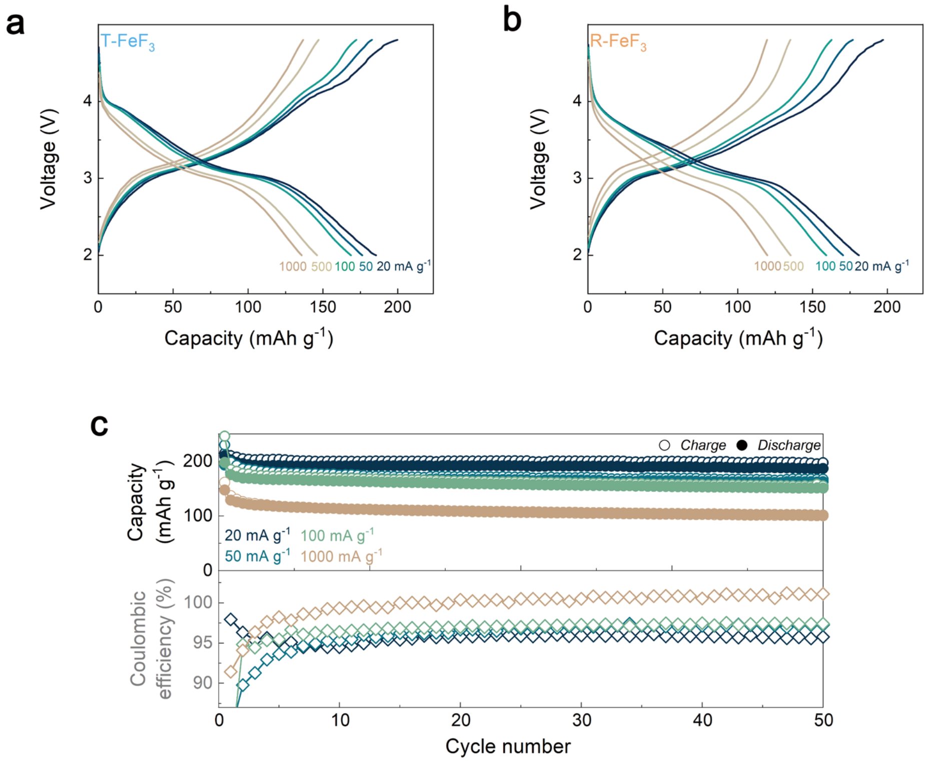

Supplementary Figure S29: Electrochemical Performance at Various Current Densities. **(a,b)** Electrochemical profile of T-FeF_3 and R-FeF_3 corresponding charge/discharge profiles for various current densities (20, 50, 100, 500, 1000 mA g^{-1}) at 25 °C in the 4.8–2.0 V voltage range. **(c)** Cycle stability and columbic efficiency of T-FeF_3 at various current densities.

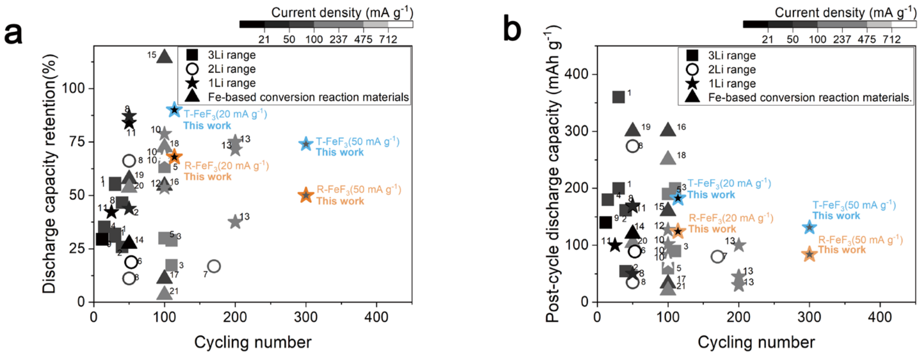

Supplementary Figure S30: Comparison of Capacity Retention in Carbon-Composited Iron Fluoride and Other Fe-Based Conversion Materials. (a, b) Comparison of capacity retention and post-cycle discharge capacity for iron fluoride materials composited with carbon and FeF_3 (rhombohedral structure) and other Fe-based conversion-reaction materials (see Supplementary Table S7).

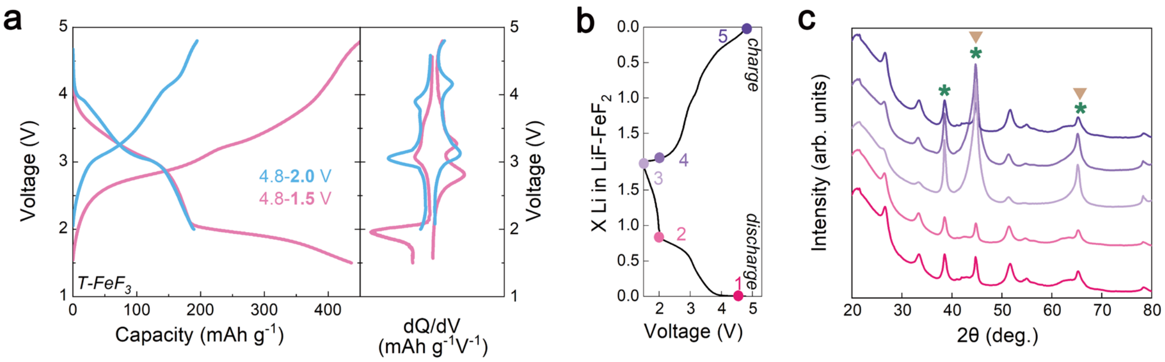

Supplementary Figure S31: Voltage Profile and Phase Evolution of LiF-FeF$_2$ in 4.8–1.5 V Ranges. (a) The 10th charge/discharge profile and differential analysis of voltage profile for LiF-FeF$_2$ in various voltage ranges, measured at 25 °C and a current density of 20 mA g^{-1}. Blue and pink are voltage profiles in the 4.8-2.0 V and 4.8-1.5 V voltage ranges, respectively. **(b)** Voltage profile for the 10th cycle depending on lithiation state in 4.8-1.5 V range, measured at 25 °C and a current density of 20 mA g^{-1}. **(c)** XRD patterns of LiF-FeF$_2$ at different Li contents in 4.8-1.5 V range. Green stars and beige inverted triangles represent LiF and Fe metal, respectively.

Supplementary Note 10. Reaction Pathway of T-FeF$_3$ Under Deep Discharge Conditions

The electrochemically induced T-FeF$_3$ from LiF-FeF$_2$ exhibits a characteristic 4 V plateau in the voltage range of 4.8-2.0 V. However, this electrochemical feature is not observed when evaluating LiF-FeF$_2$ in the deep discharge voltage range (4.8-1.5 V) (Supplementary Figure S30a). To investigate the structural changes of LiF-FeF$_2$ during deep discharge, ex situ XRD was measured at various voltages. As shown in Supplementary Figure S30c, the diffraction pattern of the tetragonal phase is observed up to point 2. However, upon discharge to 1.5 V (point 3), distinct peaks corresponding to LiF (green asterisks) and Fe metal (beige inverted triangles) are clearly observed. The conversion to Fe metal at 1.5 V discharge is further confirmed through XAS analysis. Supplementary Figure S31 displays the XANES and EXAFS spectra for the charge/discharge cycles of LiF-FeF$_2$ in the 4.8-2.0V and 4.8-1.5V ranges. The high-intensity pre-edge peak of the Fe *K*-edge in the discharged state (D-1.5V) is characteristic of Fe metal. In the EXAFS spectrum, peaks at 2.3 and 4.38 Å, corresponding to Fe metal, are prominently observed at D-1.5V. These results confirm the conversion to Fe metal under deep discharge conditions, a reaction pathway consistent with R-FeF$_3$ (Supplementary Figure S35)[13,15–17].

Interestingly, T-FeF$_3$ is not formed in the charged state of the deep discharge voltage range (C-1.5V) (Supplementary Figure S32). The Fe *K*-edge spectrum at C-1.5V does not shift back to the energy level observed at C-2.0V, and the Fe-F distance influenced by the oxidation state of Fe is 0.1 Å longer at C-1.5V compared to C-2.0V (Supplementary Figure S31). This suggests

that Fe was not oxidized as much at C-1.5V as at C-2.0V. As demonstrated in Supplementary Note 7, the formation of T-FeF_3 requires sufficient LiF splitting. However, the Fe metal produced at D-1.5V is highly stable, necessitating a substantial overpotential for LiF and Fe to react and form iron fluoride[18,19]. Therefore, the absence of distinct T-FeF_3 at C-1.5V indicates insufficient LiF splitting.

To verify this, we first performed a deep discharge and then evaluated LiF-FeF_2 in an environment with sufficient LiF splitting (4.8-2.0 V) (Supplementary Figure S36). As shown in Supplementary Figures S36d and S36e, a 4 V plateau, a characteristic feature of T-FeF_3, is observed in the 30th cycle. This shows that T-FeF_3 can form under sufficient conditions for LiF splitting, even when starting from a deep discharge. However, the electrochemical protocol that performs deep discharge first has a less pronounced 4V plateau compared to the protocol that forms T-FeF_3 first (point 3 in Fig. 5a). In addition, the discharge capacity is 172.5 mAh g^{-1} (Supplementary Figure S37), which is 19.1 mAh g^{-1} less than at point 3 in Fig. 5. Nevertheless, the capacity retention is superior to that of R-FeF_3, highlighting the importance of the T-FeF_3 formation.

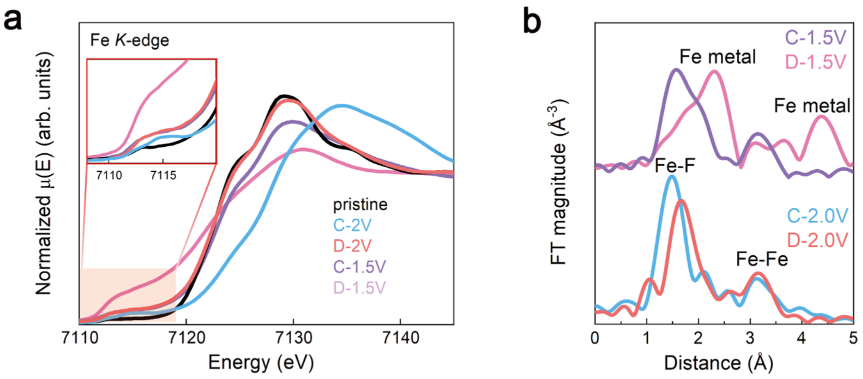

Supplementary Figure S32: Fe K-edge Spectroscopic Features in Different Voltage Windows. (a) XANES and **(b)** Fourier transform EXAFS spectra of the Fe *K*-edge for 10th charge/discharge states of LiF-FeF_2 in various voltage ranges measured at 25°C and a current density of 20 mA g^{-1}. C and D represent charging and discharging states, and the right side represents the discharging cut-off voltage.

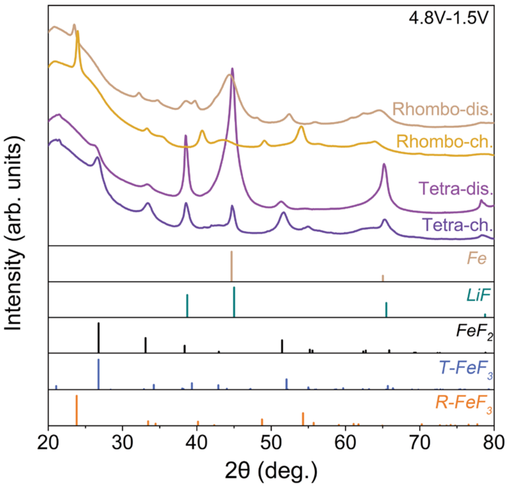

Supplementary Figure S33: Structural Evolution of LiF-FeF$_2$ and R-FeF$_3$ Between 4.8 and 1.5 V. Ex situ XRD patterns of LiF-FeF$_2$ and R-FeF$_3$ electrodes at charged/discharged states ranging from 4.8 to 1.5 V measured at 25°C and a current density of 20 mA g^{-1}.

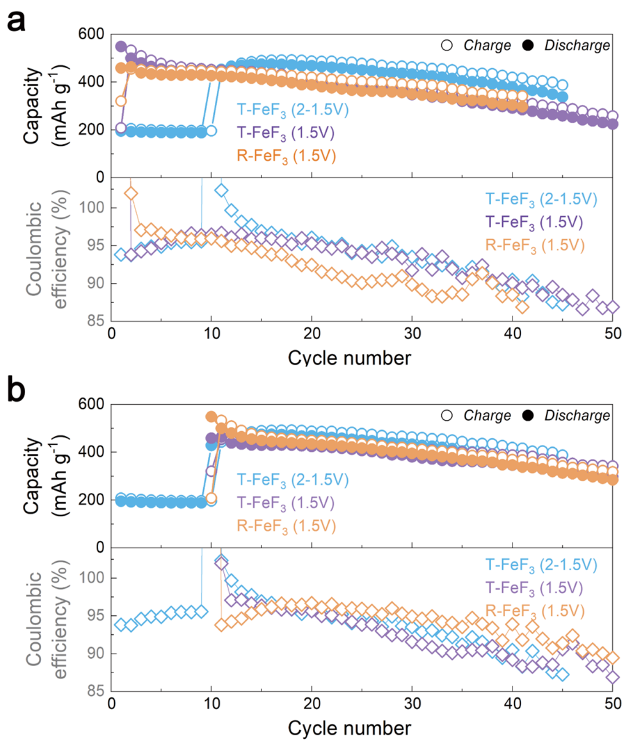

Supplementary Figure S34: Effect of Different 1.5 V Discharge Protocols on Cycling Stability. **(a)** Cycling performance of LiF-FeF_2 (purple) and FeF_3 (yellow) evaluated in the voltage range of 4.8–1.5 V is compared with the cycling performance of LiF-FeF_2 (blue) evaluated in the voltage range of 4.8–1.5 V after electrochemically forming T-FeF_3. Electrochemical formation of T-FeF_3 was performed for 10 cycles at 4.8–2.0 V, measured at 25 °C and a current density of 20 mA g^{-1}. **(b)** Cycling performance comparison of the three cases in the same voltage range of 4.8–1.5 V.

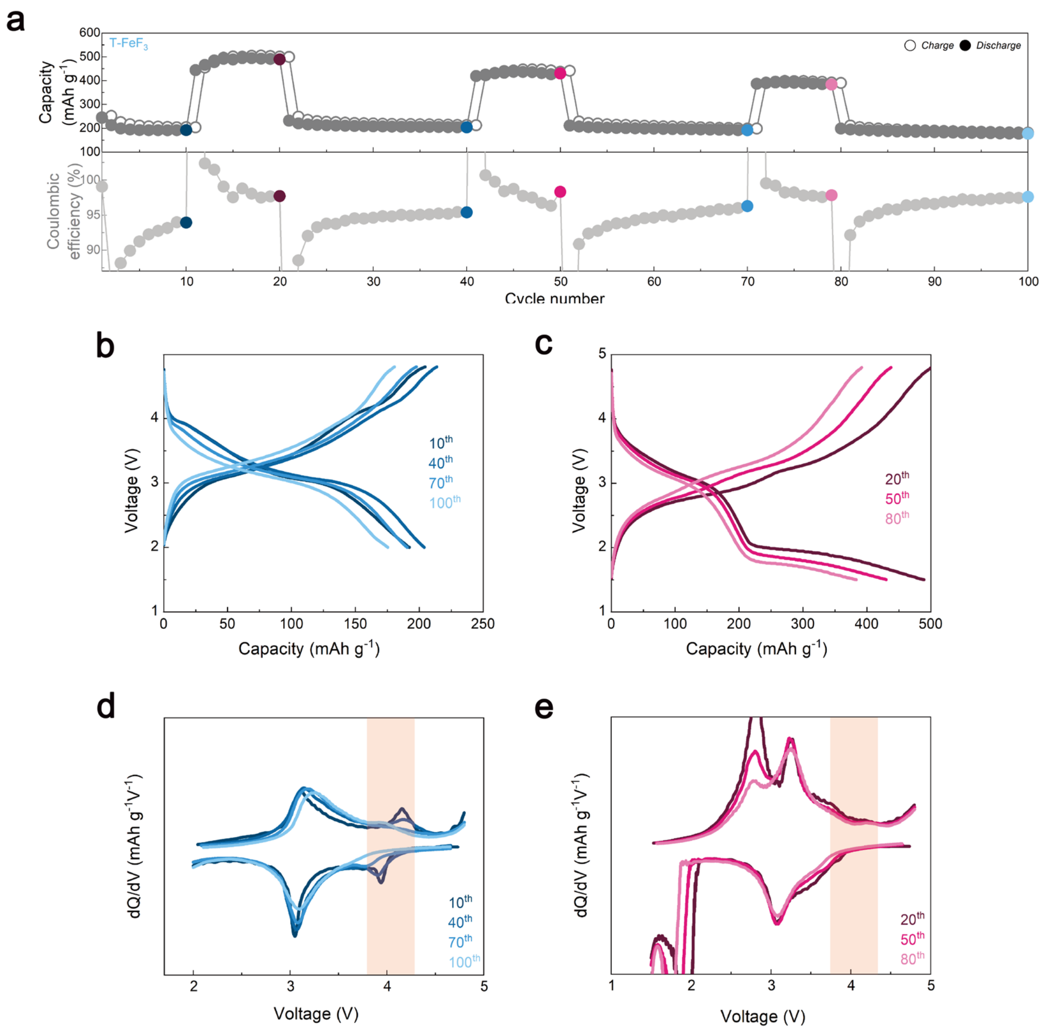

Supplementary Figure S35: Electrochemical Behavior of T-FeF_3 Under Repeated Changes in Discharge Cut-Off Voltage. (a) Specific capacity and coulombic efficiency of T-FeF_3 with repeated changed discharge cut-off voltage starting at 2 V discharge cut-off voltage, measured at 25 °C and a current density of 20 mA g^{-1}. **(b)** Electrochemical profile and **(d)** differential analysis of voltage profile at 10th, 40th, 70th, and 100th cycle (voltage range: 4.8-2.0 V). **(c)** The charge/discharge profile and **(e)** differential analysis of voltage profile at 20th, 50th, and 80th cycle (voltage range: 4.8-1.5 V).

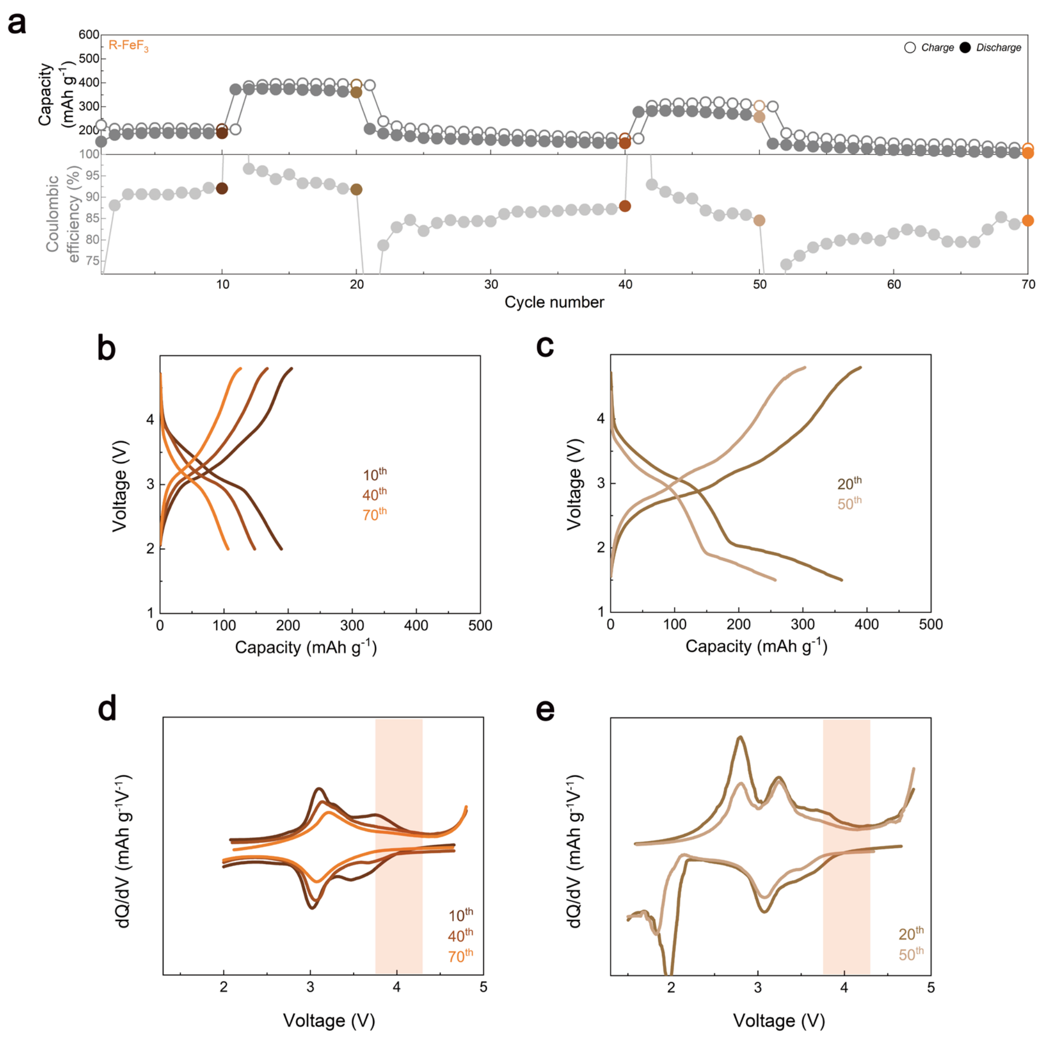

Supplementary Figure S36: Electrochemical Behavior of R-FeF_3 Under Repeated Changes in Discharge Cut-Off Voltage. (a) Specific capacity and coulombic efficiency of R-FeF_3 with repeated changed discharge cut-off voltage starting at 2 V discharge cut-off voltage, measured at 25 °C and a current density of 20 mA g^{-1}. **(b)** Electrochemical profile and **(d)** differential analysis of voltage profile at 10th, 40th, and 70th (voltage range: 4.8-2.0 V). **(c)** The charge/discharge profile and **(e)** differential analysis of voltage profile at 20th and 50th cycle (voltage range: 4.8-1.5 V).

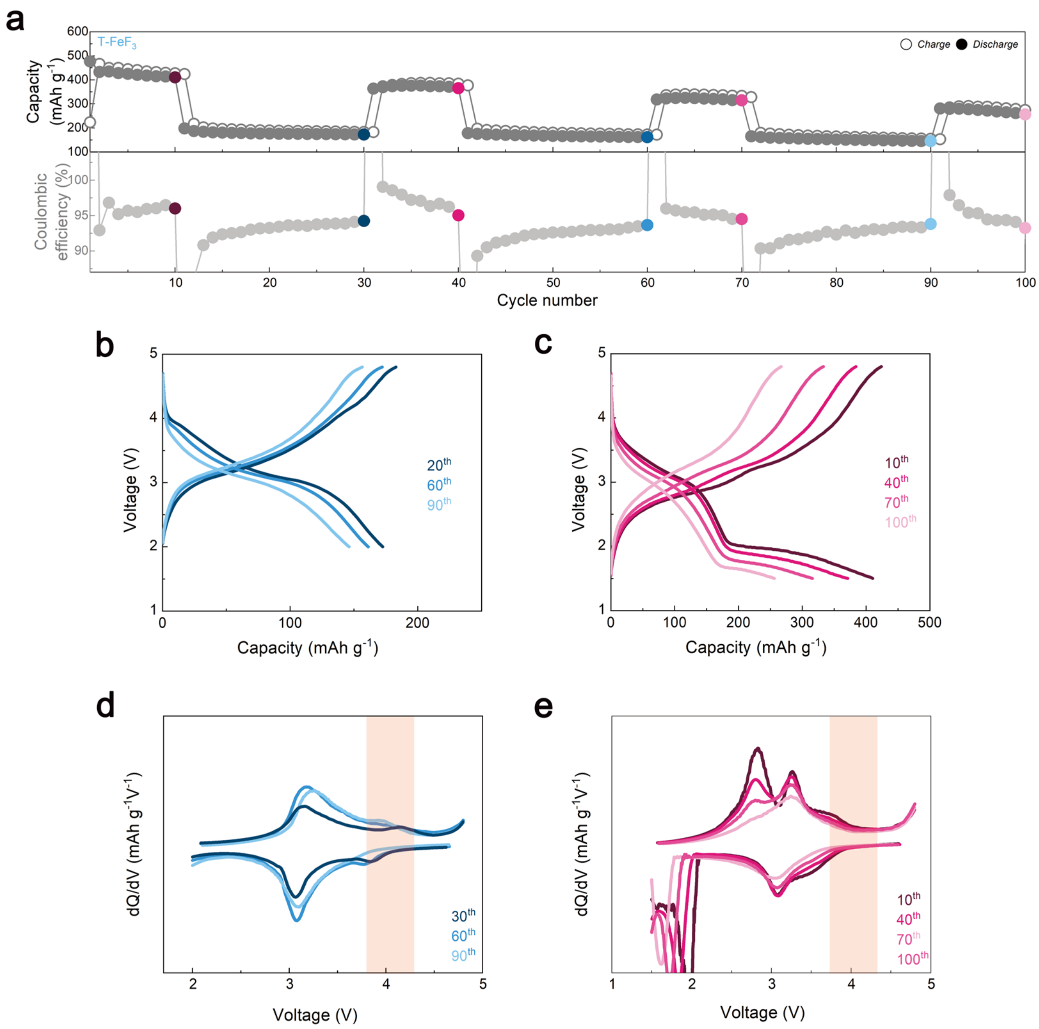

Supplementary Figure S37: Electrochemical Behavior of T-FeF_3 Under Repeated Changes in Discharge Cut-Off Voltage When First Discharged at 1.5 V. (a) Specific capacity and coulombic efficiency of T-FeF_3 with repeated changed discharge cut-off voltage starting at 1.5 V discharge cut-off voltage, measured at 25 °C and a current density of 20 mA g^{-1}. **(b)** Electrochemical profile and **(d)** differential analysis of voltage profile at 30th, 60th, and 90th (voltage range: 4.8-2.0 V). **(c)** The charge/discharge profile and **(e)** differential analysis of voltage profile at 10th, 40th, 70th, and 100th cycle (voltage range: 4.8-1.5 V).

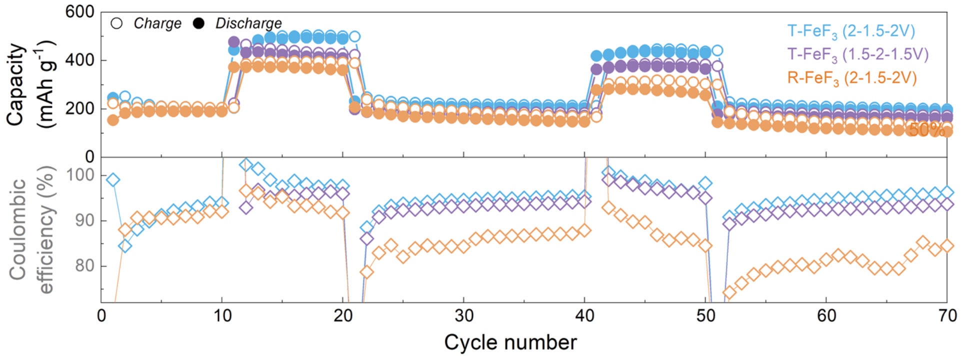

Supplementary Figure S38: Comparison of the Cycling Performance of T-FeF_3 and R-FeF_3 Under Repeated Changes in Discharge Cut-Off Voltage. The cycling behavior of T-FeF_3 and R-FeF_3 was compared under repeated variations in discharge depth, based on the capacity retention shown in Supplementary Figures S35a, S36a, and S37a. The numbers in parentheses indicate the sequence of changes in the discharge cut-off voltage. The charge voltage was fixed at 4.8 V, and measurements were conducted at 25 °C and a current density of 20 mA g^{-1}.

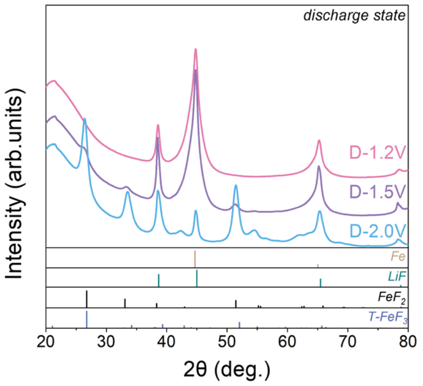

Supplementary Figure S39: Discharge Depth-Dependent Structural Evolution of LiF-FeF_2. Comparison of XRD patterns of LiF-FeF_2 according to the depth of discharge. D and numbers represent discharge and discharge cut-off voltage, respectively. XRD measurement was performed after charging and discharging until the 10th cycle where the electrochemical profile became uniform at 25 °C and a current density of 20 mA g^{-1}.

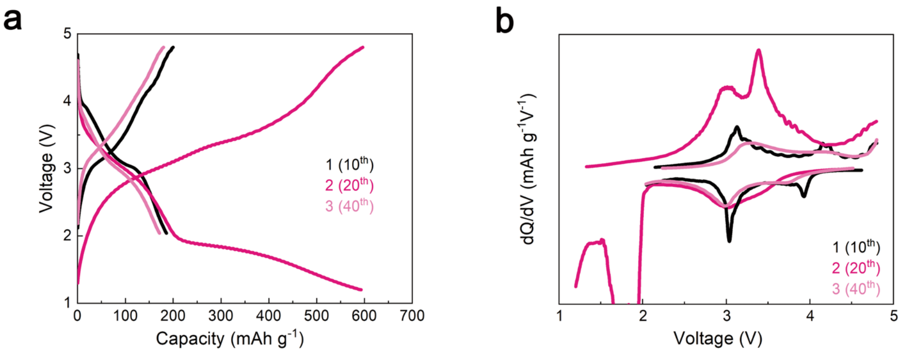

Supplementary Figure S40: Electrochemical Behavior of T-FeF_3 Under Repeated Changes in Discharge Cut-Off Voltage. (a,b) Electrochemical profile and differential analysis of voltage profile for each point in Fig 5e, measured at 25 °C and a current density of 20 mA g^{-1}.

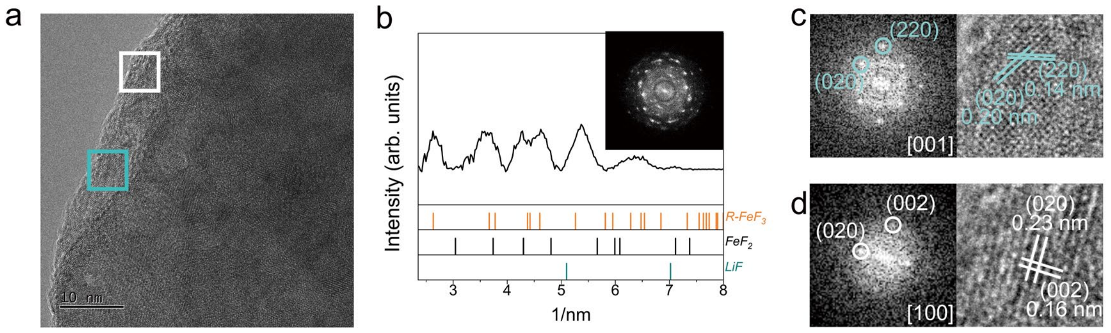

Supplementary Figure S41: TEM Analysis of R-FeF_3 at the 10th Discharge State. (a) TEM image of R-FeF3 at the 10th discharge state measured in the range of 2 V to 4.8 V at 25°C and a current density of 20 mA g^{-1}. (b) Azimuthal integration of FFT pattern for overall images. (c, d) FFT patterns at the discharge state of R-FeF_3. The green and white boxes indicate areas where the LiF and FeF_2 are predominantly present, respectively.

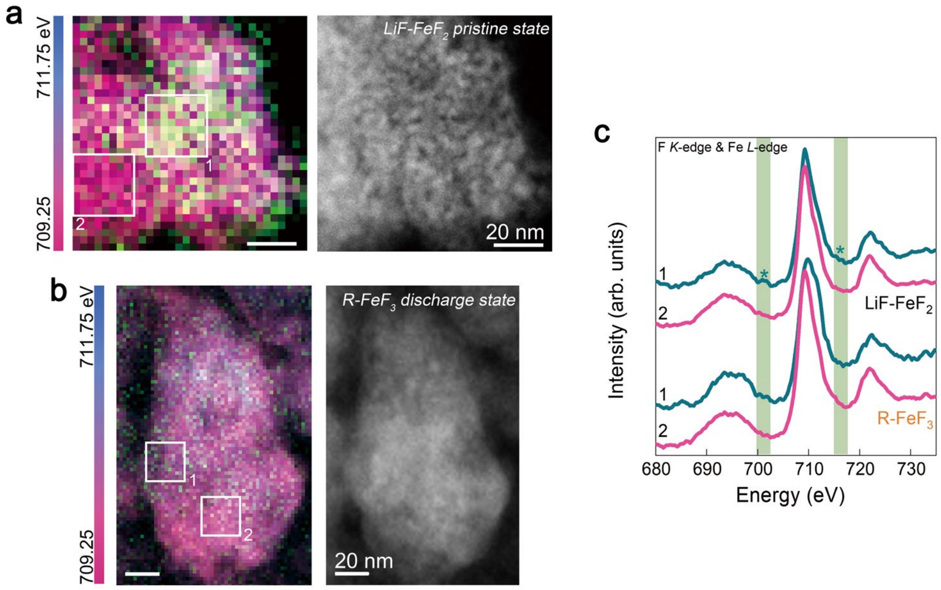

Supplementary Figure S42: Comparison of LiF Distribution Between Discharged R-FeF_3 and Pristine LiF-FeF_2. Scanning transmission electron microscopy (STEM)–electron energy loss spectroscopy (EELS) maps of (a) LiF-FeF_2 pristine state and (b) the discharged R-FeF_3, showing the spatial distribution of the Fe L_3-edge and LiF-related F K-edge signals (green). R-FeF_3 in the 10th discharged state measured in the voltage range from 2 V to 4.8 V at 25 °C and a current density of 20 mA g^{-1}. The Fe L_3-edge peak is color-coded to represent oxidation states, with blue indicating the most oxidized regions and pink indicating the most reduced regions. The LiF-related F K-edge signal is shown in green. (c) EELS spectra of the F K-edge and Fe $L_{3,2}$-edge from two regions of LiF-FeF_2 and R-FeF_3. The LiF signal is marked with asterisks (*), where region (1) corresponds to the area with the highest LiF signal intensity, and region (2) corresponds to the area with the lowest LiF signal intensity.

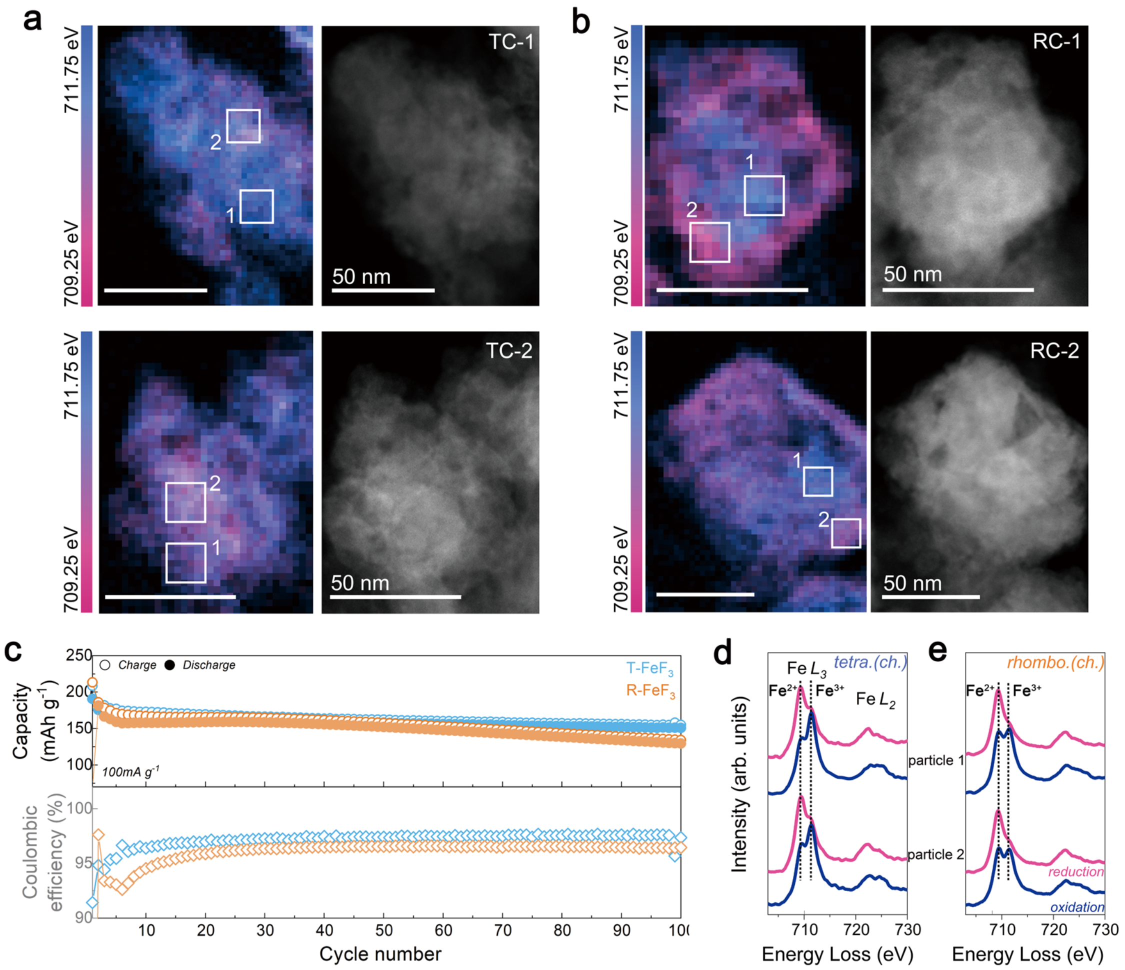

Supplementary Figure S43: Fe Oxidation State Distribution in T-FeF₃ and R-FeF₃ After Long-Term Cycling. (a,b) STEM-EELS images of T-FeF_3 and R-FeF_3 at charged state after 100th cycle (corresponding to Figure S43c) for the energy distribution of the Fe L_3-edge peak. The charge state of T-FeF_3 (TC) and R-FeF_3 (RC). The most oxidized and most reduced regions within the particle are marked 1 and 2, respectively. The scale bar in all images represents 50 nm. (c) Cycle stability and coulombic efficiency at 100 mA g^{-1} of T-FeF_3 and R-FeF_3 at 25 °C in the 4.8–2.0 V voltage range. EELS spectra of Fe $L_{3,2}$-edge peak energy of (d) TC and (e) RC.

Supplementary Table S1. Crystallographic parameters of LiF-FeF_2 nanocomposite obtained from Rietveld refinement**.**

Formula		FeF_2	LiF
Space group		$P4_2/mnm$	Fm-3m
Fract. (%)		48.82	51.18
Lattice	a (Å)	4.7105	4.0366
	b (Å)	4.7105	4.0366
	c (Å)	3.2512	4.0366
	Volume ($Å^3$)	72.142	65.77
	α (°)	90	90
	β (°)	90	90
	γ (°)	90	90
FeF_2	Atom	Fe	F
	site	2a	4f
	x	0.0000	0.1984
	y	0.0000	0.8016
	z	0.0000	0.5000
	occ.	1.0000	1.0000
LiF	Atom	Li	F
	site	4a	4b
	x	0.0000	0.0000
	y	0.0000	0.5000
	z	0.0000	0.0000
	occ.	1.0000	1.0000
Fitting indicators	R_p: 1.22	R_{wp}: 2.44	R_{exp}: 1.02 Chi2: 1.45

Supplementary Table S2. EXAFS structure parameters of the 10th charge state fitted to each structure.

structure	Path	N	ΔE (eV)	R (Å)	σ^2 (Å ^{2})	Reduced χ^2	R-factor
**Ordered Tetragonal FeF_3**	Fe-F_1	6	-2.85	1.921 ±0.003	0.0082 ±0.0006	161	0.025
	Fe-Fe_1	4	-2.85	3.690 ±0.022	0.0148 ±0.0032		
**T-FeF_3**	Fe-F_1	4	-2.79	1.926 ± 0.002	0.0032 ± 0.0003	12	0.003
	Fe-F_2	2	-2.79	2.141 ± 0.007	0.0082 ± 0.0014		
	Fe-Fe_1	2	-2.79	3.162 ± 0.004	0.0050 ± 0.0005		
	Fe-Fe_2	8	-2.79	3.688 ± 0.011	0.0253 ± 0.0018		
**R-FeF_3**	Fe-F_1	6	-2.39	1.925 ± 0.005	0.0106 ± 0.0008	224	0.037
	Fe-Fe_1	6	-2.39	3.754 ± 0.013	0.0107 ± 0.0012		

Supplementary Table S3. Crystallographic parameters of 10th charged state obtained from Rietveld refinement.

Formula			$FeF_{2.96}$	FeF_2	LiF
Space group			$P4_2/mnm$	$P4_2/mnm$	Fm-3m
Fract. (%)			67.27	7.61	25.12
Lattice	a (Å)		4.6986	4.7132	4.0380
	b (Å)		4.6986	4.7132	4.0380
	c (Å)		9.4572	3.2487	4.0380
	Volume ($Å^3$)		208.785	72.167	65.841
	α (°)		90	90	90
	β (°)		90	90	90
	γ (°)		90	90	90
$FeF_{2.96}$	Atom	Fe	Fe	F	F
	site	2a	4e	4f	8j
	x	0.0000	0.0000	0.3103	0.3177
	y	0.0000	0.0000	0.3103	0.3177
	z	0.0000	0.3221	0.0000	0.3374
	occ.	0.4240	0.8030	1.0000	1.0000
FeF_2	Atom	Fe		F	
	site	2a		4f	
	x	0.0000		0.1984	
	y	0.0000		0.8016	
	z	0.0000		0.5000	
	occ.	1.0000		1.0000	
LiF	Atom	Li		F	
	site	4a		4b	
	x	0.0000		0.0000	
	y	0.0000		0.5000	
	z	0.0000		0.0000	
	occ.	1.0000		1.0000	
Fitting indicators	R_p: 1.12	R_{wp}: 2.01		R_{exp}: 1.87	Chi2: 1.16

Supplementary Table S4. Crystallographic parameters of half-discharged state (point 2), 10th discharged state (point 3), and half-charged state (point 4) were obtained from Rietveld refinement. Each point is a point indicated in the voltage profile in Fig. 2b.

Refinement result for half-discharged state (point 2)							
Formula		$Li_{0.31}FeF_{2.96}$		FeF_2		LiF	
Space group		$P4_2/mnm$		$P4_2/mnm$		Fm-3m	
Fract. (%)		67.45		7.44		25.11	
Lattice	a (Å)	4.7012		4.7107		4.0377	
	b (Å)	4.7012		4.7107		4.0377	
	c (Å)	9.4884		3.2513		4.0377	
	Volume ($Å^3$)	209.7058		72.147		65.826	
	α (°)	90		90		90	
	β (°)	90		90		90	
	γ (°)	90		90		90	
$Li_{0.31}FeF_{2.96}$	Atom	Li	Li	Fe	Fe	F	F
	site	2a	4e	2a	4e	4f	8j
	x	0.0000	0.0000	0.0000	0.0000	0.3481	0.3171
	y	0.0000	0.0000	0.0000	0.0000	0.3481	0.3171
	z	0.0000	0.3305	0.0000	0.3305	0.0000	0.3501
	occ.	0.5600	0.0340	0.3500	0.8400	1.0000	1.0000
FeF_2	Atom		Fe			F	
	site		2a			4f	
	x		0.0000			0.1984	
	y		0.0000			0.8016	
	z		0.0000			0.5000	
	occ.		1.0000			1.0000	
LiF	Atom		Li			F	
	site		4a			4b	
	x		0.0000			0.0000	
	y		0.0000			0.5000	
	z		0.0000			0.0000	
	occ.		1.0000			1.0000	
Fitting indicators		R_p: 1.12	R_{wp}: 1.93	R_{exp}: 1.85	Chi2: 1.09		

Refinement result for 10th discharged state (point 3)			
Formula	$Li_{0.30}FeF_{2.59}$	FeF_2	LiF
Space group	$P4_2/mnm$	$P4_2/mnm$	Fm-3m
Fract. (%)	13.86	34.59	51.55

Lattice	a (Å)	4.7394		4.712		4.0361	
	b (Å)	4.7394		4.712		4.0361	
	c (Å)	9.6472		3.2514		4.0361	
	Volume ($Å^3$)	216.695		72.192		65.749	
	α (°)	90		90		90	
	β (°)	90		90		90	
	γ (°)	90		90		90	
$Li_{0.30}FeF_{2.59}$	Atom	Li	Li	Fe	Fe	F	F
	site	2a	4e	2a	4e	4f	8j
	x	0.3284	0.3122	0.0000	0.0000	0.0000	0.0000
	y	0.3284	0.3122	0.0000	0.0000	0.0000	0.0000
	z	0.0000	0.3366	0.0000	0.3134	0.0000	0.3134
	occ.	1.0000	1.0000	0.3930	0.9600	0.6020	0.0400
FeF_2	Atom		Fe			F	
	site		2a			4f	
	x		0.0000			0.1984	
	y		0.0000			0.8016	
	z		0.0000			0.5000	
	occ.		1.0000			1.0000	
LiF	Atom		Li			F	
	site		4a			4b	
	x		0.0000			0.0000	
	y		0.0000			0.5000	
	z		0.0000			0.0000	
	occ.		1.0000			1.0000	
Fitting indicators	R_p: 1.06 R_{wp}: 2.03 R_{exp}: 1.75 Chi2: 1.75						

Refinement result for half-charge state (point 4)				
Formula		$Li_{0.05}FeF_{2.83}$	FeF_2	LiF
Space group		$P4_2/mnm$	$P4_2/mnm$	Fm-3m
Fract. (%)		32.88	25.04	42.08
Lattice	a (Å)	4.7328	4.7132	4.0387
	b (Å)	4.7328	4.7132	4.0387
	c (Å)	9.6119	3.2569	4.0387
	Volume ($Å^3$)	215.301	72.349	65.873
	α (°)	90	90	90
	β (°)	90	90	90
	γ (°)	90	90	90

$Li_{0.05}FeF_{2.83}$	Atom	Li	Li	Fe	Fe	F	F
	site	2a	4e	2a	4e	4f	8j
	x	0.0000	0.0000	0.0000	0.0000	0.3295	0.3250
	y	0.0000	0.0000	0.0000	0.0000	0.3295	0.3250
	z	0.0000	0.3355	0.0000	0.3355	0.0000	0.3480
	occ.	0.0870	0.0120	0.3630	0.8780	1.0000	1.0000
FeF_2	Atom		Fe			F	
	site		2a			4f	
	x		0.0000			0.1984	
	y		0.0000			0.8016	
	z		0.0000			0.5000	
	occ.		1.0000			1.0000	
LiF	Atom		Li			F	
	site		4a			4b	
	x		0.0000			0.0000	
	y		0.0000			0.5000	
	z		0.0000			0.0000	
	occ.		1.0000			1.0000	
Fitting indicators	R_p: 1.20		R_{wp}: 2.02		R_{exp}: 1.84	Chi2: 1.20	

Supplementary Table S5. The staitistical summary for distance between cations (Li and Fe) and calculated energy of the fully delithiated state (x=0) structure (Supplementary Figure S20. (a)).

	Min	Max	Median	Mean	Band range [min, max]
Tetragonal (ordered)	3.7808	3.7819	3.6919	3.5738	[3.6939, 3.3953]
Tetragonal (disordered)	3.0965	3.6939	3.7063	3.6207	[3.7441, 3.5746]
Rhombohedral	3.0954	3.9123	3.7808	3.7812	[3.7808, 3.7819]
C2	3.6946	3.7144	3.7066	3.7048	[3.6951, 3.7144]

Supplementary Table S6. The staitistical summary for distance between cations (Li and Fe) and calculated energy of the fully lithiated state (x=1) structure (Supplementary Figure S20. (b)).

	Min	Max	Median	Mean	Band range [min, max]
Tetragonal (ordered)	2.3168	3.9934	3.4390	3.4131	[3.0374, 3.7431]
Tetragonal (disordered)	2.7776	3.9941	3.4064	3.4164	[3.0493, 3.7865]
Rhombohedral	2.5940	3.9698	3.4808	3.4284	[3.0303, 3.8576]
P312	3.3	3.7655	3.4769	3.4153	[3.0267, 3.8332]
FeF_2	2.4074	3.9989	3.5327	3.5327	[3.3, 3.7654]

Supplementary Table S7. Comparison of previously reported iron fluoride materials composited with carbon and Fe-based conversion reaction materials.

No.	ref.	Sample	Theoretical Capacity (mAh g^{-1})	Initial discharge Capacity (mAh g^{-1})	Cycling number	Current (mA g^{-1})	Post-cycle discharge capacity (mAh g^{-1})	capacity retention	Voltage range
This work		T-FeF_3	224 (1Li)	205.11	114	20	182.68	89.06%	2.0-4.8V
				178.49	300	50	128.85	72.19%	
		R-FeF_3	237 (1Li)	185.19	114	20	124.23	67.08%	
				164.74	300	50	83.89	50.92%	
1	(20)	FeF_3/C (70:25)	712 (3Li)	650	30	71.2	360	55.40%	1.0-4.5V
				620			200	32.30%	
				650			200	30.80%	
				645			360	55.80%	
2	(21)	FeF_3	712 (3Li)	210.51	40	50	54.42	25.90%	1.0-4.0V
		FeF_3/AB(85:15)		346.25			161.58	46.70%	
3	(22)	FeF_3–H-rGO	712 (3Li)	690	110	100	200	29.00%	1.0-4.5V
		FeF_3		520			90	17.30%	
4	(23)	FeF_3/AB (70:25)	712 (3Li)	510	15	71.2	180	35.30%	1.0-4.5V
5	(24)	FeF_3/C-SWNT	712 (3Li)	300	100	300	190	63.33%	1.0-4.5V
		FeF_3		200			60	30.00%	1.0-4.0V
6	(25)	FeF_3/C	475 (2Li)	170	53	20	89	52.40%	1.5-4.5V
7	(26)	bare FeF_3	475 (2Li)	598	170	100	80	13.40%	1.5-4.5V
8	(27)	FeF_3/r-GO1.7	237 (1Li)	195	50	23.7	170	87.18%	2.0–4.5 V
			475 (2Li)	414		71.2	274	66.18%	1.5–4.5 V
		FeF_3	237 (1Li)	114		23.7	50	43.86%	2.0–4.5 V
			475 (2Li)	314		71.2	35	11.15%	1.5–4.5 V
9	(28)	FeF_3	712 (3Li)	710	12	14.3	140	19.72%	1.5–4.5 V
10	(29)	FeF_3/AB (85:15)	237 (1Li)	129.3	100	237	101.9	78.80%	2.0-4.5V
				119		474	83.6	70.30%	
				105.1		1185	71.2	67.70%	
11	(30)	FeF_3/AB (70:25)	237 (1Li)	210	25	10	100	47.60%	2.0-4.5V
		FeF_3/AB-HT		200	50	10	168	84.00%	
12	(31)	FeF_3/C composite	237 (1Li)	170	100	250	127	74.71%	2.0-4.5V

No.	ref.	Sample	Theoretical Capacity (mAh g^{-1})	Initial discharge Capacity (mAh g^{-1})	Cycling number	Current (mA g^{-1})	Post-cycle discharge capacity (mAh g^{-1})	capacity retention	Voltage range
13	(32)	FCO (FeF_3@carbon nanocomposite)	237 (1Li)	120	200	474	45	37.50%	2.0-4.5V
		FCN (FeF_3@N-doped carbon nanocomposite)		140			100	71.43%	
14	(25)	C/FeOF/FeF3	-	438.3	50	20	120	27.38%	1.5-4.5V
15	(33)	LiF-FeO	274 (1Li)	140	100	50	160	114.29%	1.5-4.8V
16	(34)	FeOF	885.1 (3Li)	550	100	70	300	54.55%	1.2-4.0V
17	(35)	FeOF	885.1 (3Li)	300	100	50	33	11.00%	1.0-3.8V
18	(36)	FeOF	885.1 (3Li)	345.1	100	100	250.3	72.53%	1.3-4.0V
19	(37)	FeOF	885.1 (3Li)	520	50	50	300	57.69%	1.0-4.0V
20	(38)	FeOF@CN	885.1 (3Li)	194	50	100	104	53.61%	1.2-4.0V
21	(39)	bare FeOF	885.1 (3Li)	600	100	100	20.6	3.43%	1.2-4.0V

SUPPLEMENTARY REFERENCES

1. Vinogradov, A. S. *et al.* Low-lying unoccupied electronic states in 3d transition-metal fluorides probed by NEXAFS at the F 1s threshold. *Phys. Rev. B - Condens. Matter Mater. Phys.* **71**, 1–11 (2005).

2. Sina, M. *et al.* Structural phase transformation and Fe valence evolution in FeOxF2-x/C nanocomposite electrodes during lithiation and de-lithiation processes. *J. Mater. Chem. A* **1**, 11629–11640 (2013).

3. Vinogradov, A. S. *et al.* Low-lying unoccupied electronic states in 3d transition-metal fluorides probed by NEXAFS at the F 1s threshold. *Phys. Rev. B - Condens. Matter Mater. Phys.* **71**, (2005).

4. Cosandey, F., Su, D., Sina, M., Pereira, N. & Amatucci, G. G. Fe valence determination and Li elemental distribution in lithiated FeO 0.7F 1.3/C nanocomposite battery materials by electron energy loss spectroscopy (EELS). *Micron* **43**, 22–29 (2012).

5. GOOD, G. Core-exciton absorption in the F K absorption spectra of 3d transition-metal fluorides. Phys. *Phys. Rev. B - Condens. Matter Mater. Phys.* **37**, 10895−10897 (1988).

6. Zheng, Y. *et al.* Phase Evolution of Trirutile Li0.5FeF3 for Lithium-Ion Batteries. *Chem. Mater.* **33**, 868–880 (2021).

7. Kityakarn, S., Worayingyong, A., Suramitr, A. & Smith, M. F. Ce-doped nanoparticles of TiO2: Rutile-to-brookite phase transition and evolution of Ce local-structure studied with XRD and XANES. *Mater. Chem. Phys.* **139**, 543–549 (2013).

8. Jung, S. K. *et al.* Lithium-free transition metal monoxides for positive electrodes in lithium-ion batteries. *Nat. Energy* **2**, (2017).

9. Wei, K. *et al.* Low-Overpotential LiF Splitting in Lithiated Fluoride Conversion Cathode Catalyzed by Spinel Oxide. *Adv. Funct. Mater.* **31**, 2009133 (2021).

10. Tomita, Y. *et al.* Synthesis and charge-discharge properties of LiF-NiO composite as a cathode material for Li-ion batteries. *J. Power Sources* **329**, 406–411 (2016).

11. Tomita, Y. *et al.* Synthesis and electrochemical properties of 4LiF-NiMn2O4 composite as a cathode material for Li-ion batteries. *J. Power Sources* **354**, 34–40 (2017).

12. Dimov, N., Kitajou, A., Hori, H., Kobayashi, E. & Okada, S. Electrochemical Splitting of Lif: A New Approach to Lithium-Ion Battery Materials. *ECS Meet. Abstr.* **58**, 87–99 (2014).

13. Hua, X. *et al.* Revisiting metal fluorides as lithium-ion battery cathodes. *Nat. Mater.* **20**, 841–850 (2021).

14. Jain, A. *et al.* Commentary: The materials project: A materials genome approach to accelerating materials innovation. *APL Mater.* **1**, 011002 (2013).

15. Li, L. *et al.* Origins of Large Voltage Hysteresis in High-Energy-Density Metal Fluoride Lithium-Ion Battery Conversion Electrodes. *J. Am. Chem. Soc.* **138**, 2838–2848 (2016).

16. Li, L. *et al.* Visualization of electrochemically driven solid-state phase transformations

using operando hard X-ray spectro-imaging. *Nat. Commun.* **6**, 6883 (2015).

17. Li, L., Meng, F. & Jin, S. High-capacity lithium-ion battery conversion cathodes based on iron fluoride nanowires and insights into the conversion mechanism. *Nano Lett.* **12**, 6030–6037 (2012).

18. Wang, F. *et al.* Ternary metal fluorides as high-energy cathodes with low cycling hysteresis. *Nat. Commun.* **6**, 6668 (2015).

19. Wang, F. *et al.* Conversion reaction mechanisms in lithium ion batteries: Study of the binary metal fluoride electrodes. *J. Am. Chem. Soc.* **133**, 18828–18836 (2011).

20. Senoh, H. et al. Degradation Mechanism of Conversion-Type Iron Trifluoride: Toward Improvement of Cycle Performance. *ACS Appl. Mater. Interfaces* **11**, 30959–30967 (2019).

21. Tang, M. et al. High-Temperature Electrochemical Performance of FeF3/C Nanocomposite as a Cathode Material for Lithium-Ion Batteries. *J. Mater. Eng. Perform.* **27**, 624–629 (2018).

22. Zhao, X., Hayner, C. M., Kung, M. C. & Kung, H. H. Photothermal-assisted fabrication of iron fluoride–graphene composite paper cathodes for high-energy lithium-ion batteries. *Chem. Commun.* **48**, 9909–9911 (2012).

23. Tawa, S., Yamamoto, T., Matsumoto, K. & Hagiwara, R. Iron(III) fluoride synthesized by a fluorolysis method and its electrochemical properties as a positive electrode material for lithium secondary batteries. *J. Fluor. Chem.* **184**, 75–81 (2016).

24. Chen, T. et al. Liquid phase exfoliation of nonlayered non-van der Waals iron trifluoride (FeF3) into 2D-platelets for high-capacity lithium storing cathodes. *FlatChem* **33**, 100360 (2022).

25. Li, W. et al. The facile in situ preparation and characterization of C/FeOF/FeF3 nanocomposites as LIB cathode materials. *Ionics* **24**, 1561–1569 (2018).

26. Li, L., Zhu, J., Xu, M., Jiang, J. & Li, C. M. In Situ Engineering Toward Core Regions: A Smart Way to Make Applicable FeF3@Carbon Nanoreactor Cathodes for Li-Ion Batteries. *ACS Appl. Mater. Interfaces* **9**, 17992–18000 (2017).

27. Jung, H., Song, H., Kim, T., Lee, J. K. & Kim, J. FeF3 microspheres anchored on reduced graphene oxide as a high performance cathode material for lithium ion batteries. *J. Alloys Compd.* **647**, 750–755 (2015).

28. Martha, S. K. et al. Electrode architectures for high capacity multivalent conversion compounds: Iron (ii and iii) fluoride. *RSC Adv.* **4**, 6730–6737 (2014).

29. Liu, L. et al. Excellent cycle performance of Co-doped FeF 3/C nanocomposite cathode material for lithium-ion batteries. *J. Mater. Chem.* **22**, 17539–17550 (2012).

30. Yabuuchi, N. et al. Effect of heat-treatment process on FeF3 nanocomposite electrodes for rechargeable Li batteries. *J. Mater. Chem.* **21**, 10035–10041 (2011).

31. Lee, J. & Kang, B. Novel and scalable solid-state synthesis of a nanocrystalline FeF3/C

composite and its excellent electrochemical performance. *Chem. Commun*. **52**, 9414–9417 (2016).

32. Li, J. et al. Improved Electrochemical Performance of FeF3 by Inlaying in a Nitrogen-Doped Carbon Matrix. *ChemElectroChem* **6**, 5203–5210 (2019).

33. Jung, S. K. et al. New Iron-Based Intercalation Host for Lithium-Ion Batteries. *Chem. Mater.* **30**, 1956–1964 (2018).

34. Fan, X. et al. High energy-density and reversibility of iron fluoride cathode enabled via an intercalation-extrusion reaction. *Nat. Commun.* **9**, 2324 (2018).

35. Zhou, H. et al. Phosphorus-Doped FeOF Nanoparticle-Based Cathodes for Lithium Storage. *ACS Appl. Nano Mater*. **5**, 13444–13454 (2022).

36. Zhai, J., Lei, Z., Sun, K. & Zhu, S. MXene enabled binder-free FeOF cathode with high volumetric and gravimetric capacities for flexible lithium ion batteries. Electrochim. *Acta* **423**, 140595 (2022).

37. Lin, Y. et al. Boosting the intercalation reaction of FeOF-based cathode toward highly reversible lithium storage. *Nano Energy* **128**, 109944 (2024).

38. Li, W. et al. FeOF/TiO2Hetero-Nanostructures for High-Areal-Capacity Fluoride Cathodes. *ACS Appl. Mater. Interfaces* **12**, 33803–33809 (2020).

39. Xiao, S. et al. Versatile metal fluorides in ion battery application. *J. Mater. Chem. A* **12**, 20783–20802 (2024).
