# Supplementary material for: Guided phase transition for mitigating voltage hysteresis of iron fluoride positive electrodes in lithium-ion batteries
Source: Nat Commun. 2025 Sep 29;16:8596. doi: 10.1038/s41467-025-63676-9 (PMC12479885; doi:10.1038/s41467-025-63676-9)
Supplement: Supplementary file 3 — Description of Additional Supplementary Files [file 41467_2025_63676_MOESM3_ESM.pdf]

Description of Additional Supplementary Files

Supplementary Data 1. Structures and energies of FeF_2.

Supplementary Data 2. Structures and energies of FeF_3.

Supplementary Data 3. Structures and energies of $Li_{0.5}FeF_3$.

Supplementary Data 4. Structures and energies of $LiFeF_3$.

Supplementary Data 5. Structures and energies for site stability comparison of $Li_{0.5}FeF_3$ between 2a and 4e
